# Tissue Localization and Extracellular Matrix Degradation by PI, PII and PIII Snake Venom Metalloproteinases: Clues on the Mechanisms of Venom-Induced Hemorrhage

**DOI:** 10.1371/journal.pntd.0003731

**Published:** 2015-04-24

**Authors:** Cristina Herrera, Teresa Escalante, Mathieu-Benoit Voisin, Alexandra Rucavado, Diego Morazán, Jéssica Kele A. Macêdo, Juan J. Calvete, Libia Sanz, Sussan Nourshargh, José María Gutiérrez, Jay W. Fox

**Affiliations:** 1 Instituto de Investigaciones Farmacéuticas, Facultad de Farmacia, Universidad de Costa Rica, San José, Costa Rica; 2 Instituto Clodomiro Picado, Facultad de Microbiología, Universidad de Costa Rica, San José, Costa Rica; 3 William Harvey Research Institute, Barts and The London School of Medicine and Dentistry, Queen Mary University of London, London, United Kingdom; 4 University of Virginia School of Medicine, Charlottesville, Virginia, United States of America; 5 Instituto de Biomedicina de Valencia, Consejo Superior de Investigaciones Científicas, Valencia, Spain; Instituto Butantan, BRAZIL

## Abstract

Snake venom hemorrhagic metalloproteinases (SVMPs) of the PI, PII and PIII classes were compared in terms of tissue localization and their ability to hydrolyze basement membrane components *in vivo*, as well as by a proteomics analysis of exudates collected in tissue injected with these enzymes. Immunohistochemical analyses of co-localization of these SVMPs with type IV collagen revealed that PII and PIII enzymes co-localized with type IV collagen in capillaries, arterioles and post-capillary venules to a higher extent than PI SVMP, which showed a more widespread distribution in the tissue. The patterns of hydrolysis by these three SVMPs of laminin, type VI collagen and nidogen *in vivo* greatly differ, whereas the three enzymes showed a similar pattern of degradation of type IV collagen, supporting the concept that hydrolysis of this component is critical for the destabilization of microvessel structure leading to hemorrhage. Proteomic analysis of wound exudate revealed similarities and differences between the action of the three SVMPs. Higher extent of proteolysis was observed for the PI enzyme regarding several extracellular matrix components and fibrinogen, whereas exudates from mice injected with PII and PIII SVMPs had higher amounts of some intracellular proteins. Our results provide novel clues for understanding the mechanisms by which SVMPs induce damage to the microvasculature and generate hemorrhage.

## Author Summary

Local and systemic hemorrhage are typical manifestations of envenomings by viperid snakes. Hemorrhagic activity is due to the action of snake venom metalloproteinases (SVMPs) in the microvasculature, especially in capillaries. There are large differences in the hemorrhagic potential of SVMPs, depending on their domain composition. The present study compared PI, PII and PIII hemorrhagic SVMPs for their tissue distribution and their ability to cleave proteins of the extracellular matrix (ECM), especially those of the basement membrane (BM) that provides mechanical stability to microvessels. Observations indicate that PII and PIII SVMPs, which exert a high hemorrhagic activity, are preferentially located in microvessels, whereas PI SVMP is distributed in a more widespread fashion in the tissue. In addition, when these toxins are injected at doses that induce a similar hemorrhagic effect, they cleave type IV collagen to a similar extent, showing differences in the cleavage patterns of other ECM components, such as laminin, nidogen and type VI collagen. The analysis of the exudates resultant from the action of these SVMPs in the tissue revealed many similarities and some differences in the exudate proteomes. Overall our results indicate that hydrolysis of type IV collagen is a key event in the onset of microvessel damage, and that the ability of SVMPs to bind to microvessels greatly determines their hemorrhagic potential.

## Introduction

Zinc-dependent enzymes of the M12 reprolysin family of metalloproteinases are abundant components in the venoms of snakes, especially from species classified in the family Viperidae [1]. Snake venom metalloproteinases (SVMPs) have undergone a complex process of molecular evolution after the recruitment in the venom gland of an ADAM-like enzyme, an event that occurred before the diversification of the advanced families of the superfamily Colubroidea [2–4]. Further events included gene duplication, domain loss and neofunctionalization through mutations in regions coding for surface-exposed residues [5]. Such complex evolutionary landscape has generated a great diversity of SVMPs in snake venoms with a wide spectrum of biological activities. In addition, post-transcriptional and post-translational events further contribute to determine the final pattern of SVMPs in a particular venom [6].

On the basis of domain constitution, three main classes of SVMPs occur in viperid venoms [1]: class PI is comprised by enzymes containing only the metalloproteinase domain in the mature protein, including the canonical zinc-binding motif HEXXHXXGXXH followed by a Met-turn motif. SVMPs of the class PII present a disintegrin domain following the metalloproteinase domain; in many enzymes, this disintegrin domain is proteolytically released from its precursor [1,7]. Class PIII SVMPs comprise, in addition to the metalloproteinase domain, a disintegrin-like (Dis-like) domain followed by a cysteine-rich domain (Cys-rich). Post-translational processing of precursors of some PIII metalloproteinases results in the release of the Dis-like and Cys-rich domains (DC fragment) [1]. Further heterogeneity arises from the fact that some PII and PIII SVMPs occur as dimers, and some PIII enzymes are comprised of an additional subunit constituted by a C-type lectin-like protein, linked to the main proteinase chain by disulfide bonds [1].

These variations in domain composition have implications for the function of these enzymes and for their toxic profile. The non-metalloproteinase domains in PII and PIII SVMPs contain exosites that determine the binding of these enzymes to particular targets in the extracellular matrix (ECM), especially in microvessels, or in the plasma membrane of cells [8–14]. Immunohistochemical observations revealed a distinct pattern of distribution of PI and PIII SVMPs in the tissue [8]. In addition, the presence of these domains may prevent the inhibition of these SVMPs by the plasma inhibitor α2-macroglobulin [15,16], thus allowing them to act systemically after gaining access to the bloodstream. A potential consequence of the presence of these non-metalloproteinase domains is that PIII SVMPs generally have a greater hemorrhagic potency than PI SVMPs [17,18]. Moreover, although few PII SVMPs have been characterized in terms of hemorrhagic activity, two of them have been demonstrated as highly active hemorrhagic toxins [16,19]. Despite experimental evidence of differential binding of PI and PIII SVMPs to tissue structures *in vivo* and ECM proteins *in vitro*, a detailed comparative analysis of location of PI, PII and PIII hemorrhagic SVMPs in tissue is lacking.

The ability of hemorrhagic SVMPs to degrade basement membrane (BM) components has been known for many years, and it has been hypothesized that hydrolysis of BM proteins is a key event in the onset of microvascular damage and hemorrhage by SVMPs [20–23]. When comparing the patterns of hydrolysis of BM components *in vivo* and *in vitro* between hemorrhagic and non-hemorrhagic PI SVMPs from *Bothrops* sp venoms, a striking difference was found regarding degradation of type IV collagen, as this BM component was hydrolyzed by the hemorrhagic toxin but not by the non-hemorrhagic SVMP [22]. Since type IV collagen plays a key role in the mechanical stability of BM and hence of the capillary vessel structure [24–27], this observation is likely to have relevant functional implications regarding the mechanism of action of hemorrhagic SVMPs. It is therefore necessary to expand these studies to SVMPs of the classes PII and PIII to assess whether the pattern of hydrolysis of ECM components, particularly those of the BM, is similar to the one described for PI SVMPs or whether enzymes of different classes present different degradation patterns.

The combination of complementary analytical experimental tools is necessary to gain a deeper understanding on the mechanism of action of hemorrhagic SVMPs. In the present study we explored the patterns of tissue localization of PI, PII and PIII SVMPs using an *ex vivo* model in the cremaster muscle of mice and immunofluorescence confocal microscopy. In parallel, the patterns of BM protein degradation on skin and muscle *in vivo* were investigated by immunochemical analysis of tissue homogenates and exudates. Finally, a proteomic analysis of exudate collected from the tissue affected by the SVMPs was performed. Such proteomic analysis constitutes a ‘window’ through which details of toxin-induced tissue alterations, unobserved by more traditional histological analyses, can be detected [28,29]. Our findings reveal a distinct pattern of tissue localization of these SVMPs, with PII and PIII enzymes showing a close association with the microvasculature, in contrast to PI SVMP, which had a more widespread distribution in the tissue. Furthermore, variable patterns of degradation between the SVMPs were observed for nidogen and laminin, whereas type IV collagen was hydrolyzed to a similar extent by the three enzymes. In addition, proteomic analysis of exudate showed variations which suggest differences in the pathological effects induced by these toxins. In summary, these observations provide for a new and more complete understanding the mechanism of microvessel damage and hemorrhage induced by SVMPs.

## Methods

### Isolation of SVMPs and characterization of a new PIII from the venom of *C*. *simus*

The PI SVMP BaP1 was isolated from the venom of *Bothrops asper* as described by Gutiérrez et al. [30] and Watanabe et al. [31] by a combination of ion-exchange chromatography on CM-Sepharose, followed by affinity chromatography on Affi-gel Blue. The dimeric PII SVMP BlatH1 was purified from the venom of *Bothriechis lateralis* as described by Camacho et al. [16] by ion-exchange chromatography on DEAE-Sepharose, followed by hydrophobic interaction chromatography on Phenyl Sepharose and gel filtration on Superdex 200 10/300GL. A novel PIII SVMP was purified from the venom of adult specimens of the Central American rattlesnake *Crotalus simus*. The venom was fractionated by ion-exchange chromatography on a DEAE-Sepharose column using a BioLogic LP chromatography system (Bio-Rad). After washing the column with initial buffer (0.25 mM Tris-HCl, 2.5 mM CaCl_2_, pH 7.0), a linear gradient was developed from 0 to 0.35 M NaCl in the starting buffer. The last fraction eluted, consisting mainly of a 55 kDa hemorrhagic toxin, was further purified by gel filtration chromatography on a SuperdexTM 200 10/300GL (GE Healthcare, LifeSciences) column (10 x 300 mm) previously equilibrated with 0.05 M Tris-HCl, 5 mM CaCl_2_, pH 5.8, buffer using an ÄKTA FPLC (GE Healthcare, LifeSciences).

Homogeneity and molecular mass of the *C*. *simus* SVMP were determined by SDS-polyacrylamide gel electrophoresis (SDS-PAGE), run under reducing and non-reducing conditions [32]. SDS-PAGE electrophoresis was performed on 15% Tris-HCl polyacrylamide gel and staining was performed, either with Coomassie Brilliant Blue for total protein, or with Pro-Q Emerald 300 Glycoprotein Stain Kit (Molecular Probes) for detection of carbohydrates. Proteolytic activity was assessed on azocasein (Sigma) as described by Wang et al. [33]. Hemorrhagic activity was evaluated by injecting various amounts of the SVMP intradermally in mice and measuring, after 2 h, the diameter of the hemorrhagic lesion in the internal side of the skin [34]. The Minimum Hemorrhagic Dose (MHD) corresponds to the amount of enzyme that induces a hemorrhagic spot of 10 mm diameter 2 h after injection. In addition, the ability of this enzyme to induce pulmonary hemorrhage was assessed by injecting 50 μg of the enzyme by the intravenous route in mice. One hour after injection, mice were sacrificed by an overdose of xylazine and ketamine, and lungs were dissected out and routinely processed for embedding in paraffin and staining with hematoxylin-eosin.

For tryptic peptide mapping and internal peptide sequence determination, purified PIII-SVMP was excised from a Coomassie Brilliant Blue-stained SDS-PAGE and subjected to automated reduction (10 mM dithiothreitol) and alkylation (50 mM iodacetamide), followed by overnight trypsin digestion (66 ng/μL of sequencing-grade porcine trypsin (Promega) in 25 mM ammonium bicarbonate, 10% acetonitrile; 0.25 μg/sample) in a ProGest^TM^ Protein Digestion Workstation (Genomics Solutions) following manufacturer's instructions. For peptide sequencing, the protein digest mixture was loaded in a nanospray capillary column and subjected to electrospray ionization (ESI) mass spectrometric analysis using a QTrap^TM^ 2000 mass spectrometer (Applied Biosystems) equipped with a nanospray source (Protana, Denmark). Doubly- or triply-charged ions were analyzed in Enhanced Resolution MS mode and the monoisotopic ions were fragmented using the Enhanced Product Ion tool with Q_0_ trapping. Enhanced Resolution was performed at 250 amu/s across the entire mass range. Settings for collision-induced dissociation MS/MS experiments were as follows: Q1- unit resolution; Q1-to-Q2 collision energy—30–40 eV; Q3 entry barrier—8 V; LIT (linear ion trap) Q3 fill time—250 ms; and Q3 scan rate—1000 amu/s. Product ion spectra were interpreted manually.

The complete amino acid sequence of *C*. *simus* PIII SVMP was determined by a combination of tryptic peptide MS/MS sequencing and cDNA cloning from a venom gland cDNA library previously constructed and used for profiling the venom gland transcriptomes of Costa Rican snakes by 454 pyrosequencing [35]. The amplification mixture contained, in a final volume of 50 μL: 1 μL of cDNA library; 1 μL of a 10 μM stock solution of each, forward (Fw: 5-AAC CCC TTC AGA TTC GTT GAG-3´) and reverse (Rv: 3’-ATA GGC TGT AGC CAC ATC AAC-5') primers derived from the amino acid sequences NPFRFVE and VDVATAY, respectively, determined by *de novo* MS/MS tryptic ion sequencing, these amino acid sequences are highly conserved in the N- and C-termini of PIII-SVMPs, respectively; 0.25 μL of DNA polymerase (GoTaq, Promega); 1 μL of 10 mM dNTPs; 2 μL of 25 mM MgCl_2_; 5 μL of 5x buffer; and 13.75 μL of DNAse-free deionized water. The PCR protocol included initial denaturation at 94°C for 10 min, followed by 35 cycles of denaturation (10 s at 94°C), annealing (45 s at 55°C) and extension (120 s at 72°C), and a final extension for 7 min at 72°C. The amplified fragment was purified from an agarose gel using the Illustra GFX PCR DNA and Gel Band Purification kit (GE Healthcare) and cloned in a pCR-XL-TOPO vector (Invitrogen). *E*. *coli* DH5 cells (Novagen, Madison, WI, USA) were transformed by electroporation using an Eppendorf 2510 electroporator following the manufacturer´s instructions. Positive clones, selected by growing the transformed cells in Luria-Broth (LB) medium containing 10 μg/ml ampicillin, were confirmed by PCR amplification using the above primers, and the PCR-amplified fragments were sequenced using an Applied Biosystems model 377 DNA sequencer.

### Ethics statement

All *in vivo* experiments were performed in CD-1 mice. The experimental protocols involving the use of animals in this study were approved by the CICUA (University of Costa Rica) and meet the International Guiding Principles for Biomedical Research Involving Animals (CIOMS).

### Experiments *ex vivo*

#### Immunolocalization of SVMPs in muscle tissue

The mouse cremaster muscle was used to study the distribution and immunolocalization of the SVMPs because it is possible to obtain high resolution images of longitudinal blood vessels by confocal microscopy due to the transparency and thinness of this tissue. Groups of three male mice were killed by cervical dislocation and the cremaster muscle was dissected out. The isolated muscles were incubated in PBS for 15 min with either BaP1 (PI, 30 μg), BlatH1 (PII, 3.5 μg) or CsH1 (PIII, 15 μg) SVMPs labeled with Alexa Fluor 647 according to the Microscale Protein Labeling Kit (Molecular Probes A30009). These doses were selected as to induce a hemorrhage in the cremaster muscle of similar intensity to that described previously by intravital microscopy for the PI SVMP BaP1 [36]. Control tissues were incubated with unlabeled toxins. After incubation, tissues were washed with PBS and fixed with 4% paraformaldehyde in PBS for 30 min at 4°C. Fixed whole tissues were incubated for 4 h at room temperature in blocking and permeabilization solution (12.5% goat serum, 12.5% fetal bovine serum and 0.5% Triton X-100 in PBS). Then, the tissues were immunostained overnight at 4°C with rabbit anti-collagen type IV polyclonal antibody at a dilution of 1:100 (Abcam ab19808), and Cy3-labeled mouse anti-actin α smooth muscle monoclonal antibody at a dilution of 1:200 (clon 1A4, Sigma C6198) to visualize the vascular basement membrane and smooth muscle/pericytes, respectively. After three washes in PBS, tissues were incubated for 4 h at 4°C with goat anti-rabbit polyclonal antibody labeled with Alexa Fluor 488 at a dilution of 1:200 (Invitrogen A11034). In order to ascertain whether labeling of SVMPs had an effect on their enzymatic activity, proteolytic activity of the labeled enzymes was quantified on gelatin with the EnzChek Gelatinase Assay Kit (Molecular Probes E-12055) at different times during 24 h.

Immunostained tissues were mounted on glass slides and visualized using a Zeiss LSM 5 Pascal laser-scanning confocal microscope (Carl Zeiss Ltd) incorporating a 10X objective (numerical aperture 0.3) and 63X oil objective (numerical aperture 1.4). At least four images of post-capillary venules (PCV), arterioles and capillaries per tissue were taken in 3 dimensions at a resolution of 1,024 × 1,024 dpi corresponding to a voxel size of 0.14 × 0.14 × 0.38 μm in the X × Y × Z plans, respectively using the 63X objective. Three-dimensional reconstitution of the images and analysis of co-localization of the SVMPs with collagen IV were carried out using IMARIS x64 7.4.2 image analysis software, which employs the approach developed by Costes et al. [37]. A region of interest (ROI) in the pixel intensity of 10 was defined to calculate the threshold for each label with the automatic function of the software. The program IMARIS analyzes the intensity of each label by voxels defined as a prism with the pixel in the base and the thickness of the confocal section in the height. The results of co-localization were expressed as the percentage of material co-localized which takes into account the number of voxels co-localized and the Pearson´s correlation coefficient, which reflects the correlation between intensities in the co-localized voxels. The Pearson’s coefficient varies between +1 and -1, where values near 1 indicate a direct correlation, and values near 0 indicate no correlation. The percentage of co-localization may overestimate the extent of co-localization, while the Pearson’s coefficient may underestimate the extent of co-localization since it takes into account that the intensity of the two labels varies together. Hence, it is important to analyze both values in co-localization studies.

### Experiments *in vivo*

#### Immunochemical detection of ECM proteins in the skin and exudate

Groups of five mice were injected intradermally in the ventral abdominal region with either BaP1 (PI, 75 μg), BlatH1 (PII, 1.5 μg) or CsH1 (PIII, 35 μg) SVMPs, dissolved in 100 μL of PBS. These doses were selected as to induce a similar hemorrhagic area in the injected skin, since these enzymes have highly different hemorrhagic activity, i.e. Minimum Hemorrhagic Doses [16,30], [this work]. The control group received 100 μL of PBS alone. After 15 min, mice were sacrificed by CO_2_ inhalation, their skin was removed, and an area of 12 mm diameter in the site of the injection was dissected out. In order to prepare a pool, all skin fragments from each treatment were combined the placed in liquid nitrogen and pulverized until fine particles were obtained. Each pool was resuspended in 1.5 mL of extraction buffer (25 mM Tris-HCl, 150 mM NaCl, 8 M urea, 40 mM EDTA, 1% Triton X-100, 0.1% SDS, pH 7.4) with a tablet of protease inhibitor cocktail (Roche) per 10 mL of buffer. One hour after incubation under stirring at 4°C, samples were centrifuged at 5,200 g for 5 min and the supernatant was diluted 1:2 with water and stored at -70°C until Western blot analysis was performed.

In another set of experiments, groups of five mice were injected in the right gastrocnemius with either BaP1 (PI, 75 μg), BlatH1 (PII, 3 μg) or CsH1 (PIII, 50 μg) SVMPs, dissolved in 50 μL of PBS. These doses were selected as they induce a similar extent of hemorrhagic activity in muscle. After 15 min of injection, mice were sacrificed by CO_2_ inhalation, and a 5 mm incision was made with a scalpel in the skin overlying the injected muscle. Immediately, the sectioned skin was opened and a heparinized capillary tube was introduced under the skin to collect the wound fluid [28]. An approximate volume of 20–50 μL of exudate was collected from each mouse. Exudate samples were then pooled and lyophilized.

For immunoblotting, 10–20 μL of each skin homogenate sample, or 100 μg protein of each exudate sample, were separated under reducing conditions on 4–15% Tris–HCl SDS-PAGE gradient gels, and transferred to nitrocellulose membranes. Immunodetection was performed by incubating the membranes overnight at 4°C under stirring with either rabbit anti-collagen type VI polyclonal antibody at a dilution of 1:2,000 (Millipore AB7821), rabbit anti-collagen type IV polyclonal antibody at a dilution of 1:1,000 (Abcam ab6586), rabbit anti-laminin polyclonal antibody at a dilution of 1:500 (Abcam ab11575), or rabbit anti-nidogen 1 polyclonal antibody at a dilution of 1:3,000 (Abcam ab14511). The anti-GAPDH antibody at a dilution of 1:1,000 (Abcam ab9485) was used as loading control for immunoblotting of the skin homogenates samples. The reaction was detected using an anti-rabbit peroxidase antibody at a dilution of 1:10,000 (Jackson ImmunoResearch) and the chemiluminescent substrate Lumi-Ligth (Roche). The images were obtained with the ChemiDoc XRS+ System (BioRad) and the analysis was performed with the ImageLab software (BioRad).

### Analysis of the proteomics of exudates

Lyophilized wound exudate samples were re-suspended in water and protein quantification was performed using micro BCA protein assay kit (Thermo Scientific) Twenty micrograms of protein were then resuspended in Laemmli buffer, applied to a 12% precast electrophoresis gel (Bio-Rad), separated, and stained with Coomassie Brilliant Blue. Gel lanes were cut in ten equal size slices. Gel slices were destained for 3 h and the proteins reduced (10 mM DTT) and alkylated (50 mM iodoacetamide) at room temperature. Gel slices were washed with 100 mM ammonium bicarbonate, dehydrated with acetonitrile and dried in a speed vac. Hydration of the slices was performed with a solution of Promega modified trypsin (20 ng/μL) in 50 mM ammonium bicarbonate for 30 min on ice. Excess trypsin solution was removed and the digestion was carried on for an additional 18 h at 37°C. Tryptic peptides were twice extracted from gel slices with 30 μL of a 50% acetonitrile/5% formic acid solution. The combined extracts were dried to a volume of 15 μL for mass spectrometric analysis. LC/MS/MS was performed using a Thermo Electron Orbitrap Velos ETD mass spectrometer system. Analytical columns were fabricated in-house by packing 0.5 cm of irregular C18 Beads (YMC Gel ODS-A, 12 nm, I-10-25 um) followed by 7.5 cm Jupiter 10 μm C18 packing material (Phenomenex, Torrance, CA) into 360 x 75 μm fused silica (Polymicro Technologies, Phoenix, AZ) behind a bottleneck. Samples were loaded directly onto these columns for the C18 analytical runs. Aliquots of 7 μL were loaded onto the column for each analysis and eluted into the mass spectrometer at 0.5 μL/min using an acetonitrile/0.1M acetic acid gradient (2–90% acetonitrile over 1 h). The instrument was set to Full MS (m/z 300–1600) resolution of 60,000 and programmed to acquire a cycle of one mass spectrum followed by collision-induced dissociation (CID) MS/MS performed in the ion trap on the twenty most abundant ions in a data-dependent mode. Dynamic exclusion was enabled with an exclusion list of 400 masses, duration of 60 seconds, and repeat count of 1.The electrospray voltage was set to 2.4 kV, and the capillary temperature was 265°C.

Peak lists were generated from the raw data using the Sequest search algorithm in Proteome Discoverer 1.4.1 against the Uniprot Mouse database from July 2014. Spectra generated were searched using carbamidomethylation on cysteine as a fixed modification, oxidation of methionine as a variable modification, 10 ppm parent tolerance and 1 Da fragment tolerance. All hits were required to be fully tryptic. The results from the searches were exported to Scaffold (version 4.3.2, Proteome Software Inc., Portland, OR). Scaffold was used to validate MS/MS based peptide and protein identifications and to visualize multiple datasets in a comprehensive manner. Protein identifications were filtered using Xcorr cutoff values dependent on charge state (+1>1.8, +2>2.2, +3>2.5 and +4>3.5). Confidence of protein identification in Scaffold is displayed as a Probability Legend with green coloration indicative of over 95% confidence and yellow as 80% to 94% confidence. Relative quantization of proteins was accomplished by summing all data from the 10 gel slices for a particular sample in Scaffold and then displaying the Quantitative Value from the program. This number gives an average total of non-grouped spectral counts for a protein divided by the total non-grouping spectral counts for the 10 mass spectral runs from the gels slices from each lane (http://www.proteomesoftware.com/). This format of presentation allows for a relative quantitative comparison between a specific protein from different samples and to a certain degree gives some measure of relative abundance between proteins generated from the mass spectrometric analysis of the 10 gel slices for a particular exudate sample. Portions of the data were further analyzed manually to determine if mass spectra were derived from proteins migrating in the gel at their expected molecular mass or at a lower mass.

## Results

### Characterization of a new PIII SVMP from the venom of *C*. *simus*

**Fig 1:**
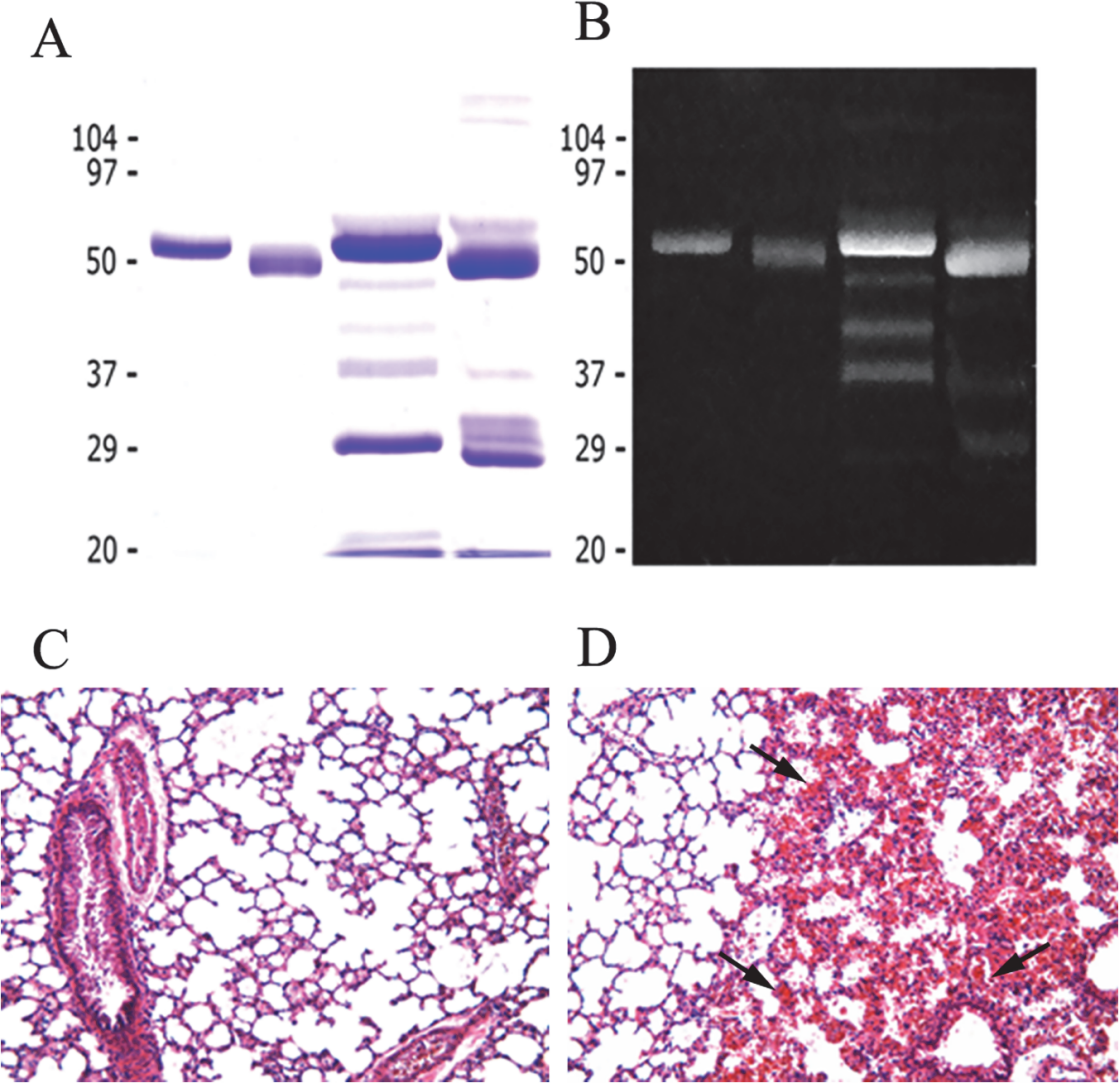
(A), (B) SDS-PAGE of *Crotalus simus* PIII SVMP and crude venom. Samples were run on a 12% gel, and stained with (A) Coomassie blue or (B) Pro-Q Emerald 300 glycoprotein stain (Molecular probes). Lane 1: SVMP, reducing conditions; lane 2: SVMP, non-reducing conditions; lane 3: *Crotalus simus* venom, reducing conditions; lane 4: *Crotalus simus* venom, non-reducing conditions. The P-III metalloproteinase is a major component of the venom; it is glycosylated and has a molecular mass of 55 kDa. (C) and (D) Light micrographs of sections of lung tissue from mice injected intravenously with either saline solution (C) or 100 μg of *C. simus* PIII SVMP. Mice were sacrificed one h after injection and tissue samples were obtained and routinely processed for embedding in paraffin and further staining with hematoxylin-eosin. Notice prominent hemorrhage in the pulmonary tissue in (D) (arrow). 125 X.

Through a combination of ion-exchange chromatography on DEAE-Sephadex and gel filtration, a novel hemorrhagic SVMP was purified to homogeneity from the venom of adult specimens of the Central American rattlesnake *C*. *simus* (Fig 1A and 1B). This SVMP is hereby named CsH1. It is a monomeric glycosylated protein with a molecular mass of 55 kDa (Fig 1A and 1B). The cDNA-deduced amino acid sequence was identified by BLAST analysis as GenBank accession number DQ164403 (the species *Crotalus simus* was previously named *Crotalus durissus durissus*), and various tryptic peptide ion sequences gathered by *de novo* MS/MS sequencing of an in-gel digested Coomassie Brilliant Blue-stained SDS-PAGE band of the purified PIII SVMP (Table 1) confirmed the assignment. The enzyme possesses a zinc metalloproteinase domain, a disintegrin-like domain and a cysteine-rich domain, hence corresponding to a PIII SVMP. The SVMP has proteolytic activity on azocasein and was hemorrhagic using the mouse skin assay, with a MHD of 2.2 μg. Both proteolytic and hemorrhagic activities were abrogated by incubation with EDTA. The enzyme was also able to induce hemorrhage in lungs after intravenous injection (Fig 1C and 1D); thus it induces both local and systemic hemorrhage.

**Table 1:** Tryptic peptide ion sequences obtained by *de novo* CID-MS/MS sequencing of *Crotalus simus* PIII SVMP.

m/z	Z	Amino acid sequence
275.3	2+	CADGK
291.8	2+	JYCK
354.7	2+	SGTECR
359.8	2+	TDJJTR
401.3	2+	GMVJPGTK
417.3	2+	NNDDJDK
467.8	2+	ZKYNPFR
526.3	2+	GNYYGYCR
615.8	2+	DNSPGQNNPCK
649.9	2+	MFYSNEDEHK
760.8	3+	YMYJHVAJVGJEJWSNEDK
766.4	2+	VJGJAYVGSMCHPK
801.3	2+	MYEJANTVNDJYR
776.2	2+	VCSNGHCVDVATAY
885.4	2+	SGSQCGHGDCCEQCK
684.6	3+	JTVKPEAGYTJNAFGEWR
869.8	3+	ZKYNPFRFVEJVJVVDKAMVTK
926.3	3+	ASMSECDPAEHCTGQSSECPADVFHK

J, Isoleucine (I) or Leucine (L); Z, pyroglutamic acid (2-oxo-pyrrolidone carboxylic acid). Cysteine residues (C) are carbamidomethylated.

### Inmunolocalization of SVMPs in the tissue

**Fig 2:**
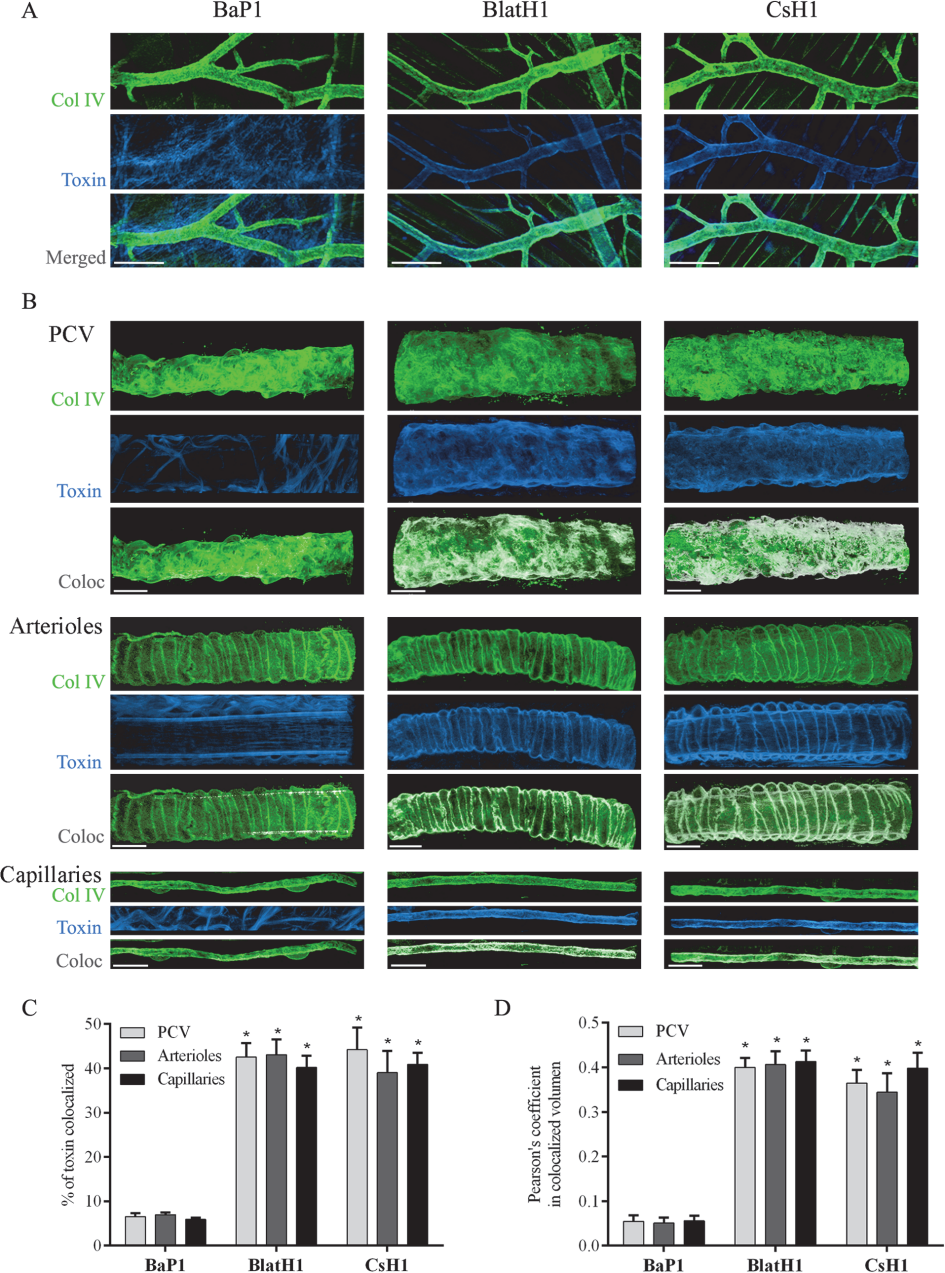
Immunolocalization of SVMPs with vascular basement membrane on cremaster muscle *ex vivo*. Isolated cremaster muscles were incubated for 15 min with equi-hemorrhagic amounts of either BaP1 (PI, 30 μg), BlatH1 (PII, 3.5 μg) or CsH1 (PIII, 15 μg) SVMPs labeled with Alexa Fluor 647 (blue). Control tissues were incubated with the SVMPs without labeling and no fluorescence was detected. Whole tissues were fixed with 4% paraformaldehyde and immunostained with anti-collagen IV following the secondary antibody labeled with Alexa Fluor 488 (green). Tissues were visualized in a Zeiss LSM 5 Pascal laser-scanning confocal microscope. Three-dimensional reconstitution of the images and analysis of co-localization were carried out with the IMARIS x64 7.4.2 software as described in Methods. (A) Distribution of the SVMPs in the cremaster muscle tissue. Scale bar represents 150 μm. (B) White areas represent co-localization of the SVMPs (blue) with collagen IV (green) of vascular basement membrane in PCV, arterioles, and capillaries. Scale bar represents 20 μm. Results are expressed as the mean ± SEM of (C) percentage of material of SMVPs co-localized with collagen IV of vascular basement membrane, and (D) Pearson´s correlation coefficient of at least four vessels type per tissue (n = 3). *p<0.001 when compared with BaP1 (PI) SVMP for post-capillary venules (PCV), arterioles, and capillaries.

When equi-hemorrhagic amounts of either PI, PII or PIII SVMPs were incubated for 15 min with the isolated cremaster muscles *ex vivo*, a clear difference in the distribution of the toxins was observed (Fig 2A). BlatH1 (PII) and CsH1 (PIII) SVMPs were preferentially localized in the basement membrane of blood vessels, as evidenced by co-localization with collagen IV. In contrast, localization of BaP1 (PI) SVMP was observed widespread in the tissue and to a lesser extent in the vascular basement membrane (Fig 2A). No fluorescence was detected in the control tissues incubated with unlabeled SVMPs. This localization of BlatH1 (PII) and CsH1 (PIII) SVMPs was noted in post-capillary venules (PCV) and also in arterioles and capillaries (Fig 2B). The proteolytic activity on gelatin of the three SVMPs after labeling with Alexa Fluor 647 was between 75–85% as compared to control unlabeled enzymes, thus indicating that labeled SVMPs remained functionally active.

The analysis of co-localization (Fig 2C and 2D) showed a higher percentage of co-localization (around 40%) and Pearson’s coefficient (around 0.4) for BlatH1 (PII) and CsH1 (PIII) SVMPs with collagen IV of the vascular basement membrane in PCV, arterioles, and capillaries as compared with BaP1 (PI) SVMP (p<0.001). No significant differences were observed between co-localization of PII and PIII SVMPs with collagen IV in the basement membrane of arterioles, capillaries, and PCV. Moreover, no significant differences were observed between co-localization of each SVMPs in the basement membrane of arterioles, capillaries, and PCV.

### Immunochemical analysis of BM-associated proteins in the skin and in exudates

**Fig 3:**
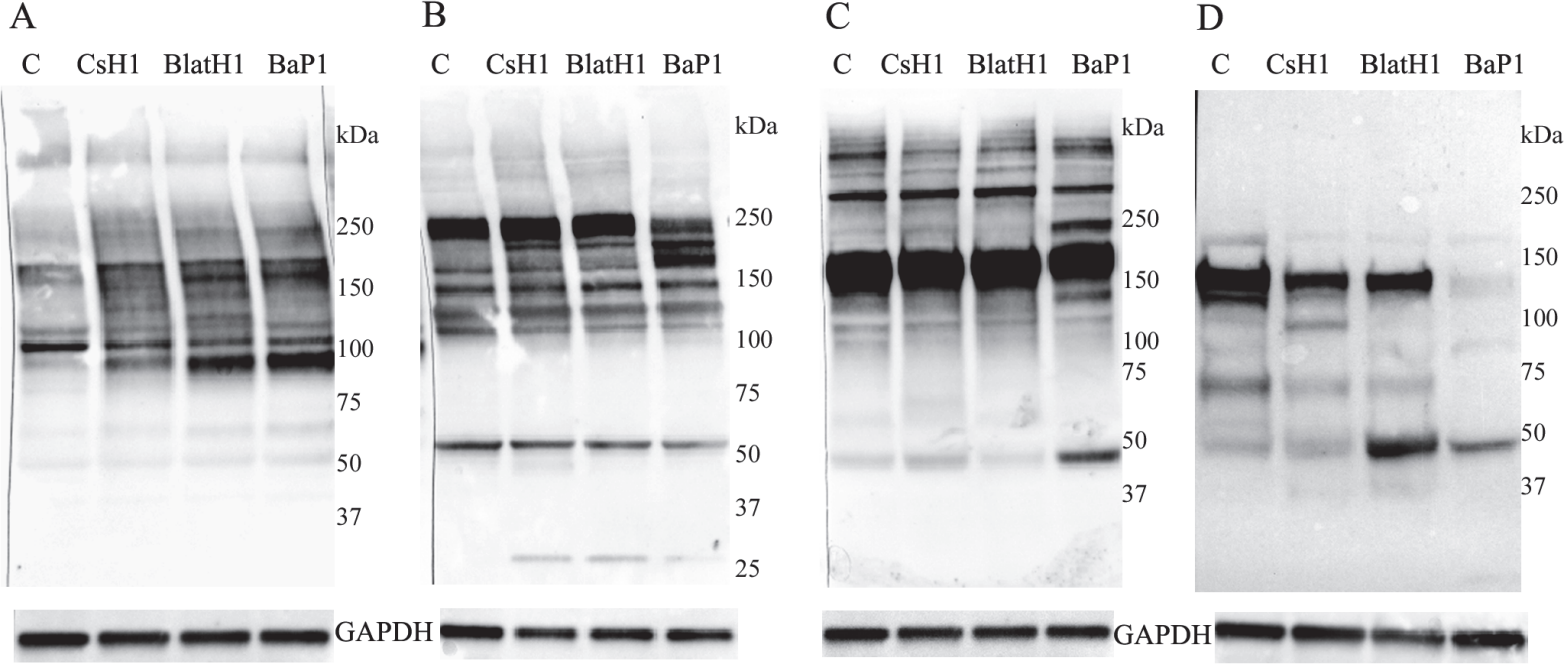
Western blot analysis of basement membrane components in skin homogenates. Groups of five mice were injected by intradermal route in the ventral abdominal region with either BaP1 (PI, 75 μg), BlatH1 (PII, 1.5 μg), CsH1 (PIII, 35 μg) SVMPs or PBS (lane C). After 15 min, mice were sacrificed, their skin was removed, and an area of 12 mm diameter was dissected out. Tissues of the same group were homogenized and centrifuged, and the supernatant collected. Then, 10–20 μL of each skin homogenate sample were separated under reducing conditions on 4–15% Tris–HCl SDS-PAGE gradient gels, and transferred to nitrocellulose membranes. Immunodetection was performed with (A) anti-collagen type IV, (B) anti-collagen type VI, (C) anti-laminin, and (D) anti-nidogen 1. The anti-GAPDH antibody was used as loading control. The reaction was detected using an anti-rabbit peroxidase antibody and a chemiluminescent substrate. Images were obtained with the ChemiDoc XRS+ System (BioRad).

Hemorrhagic lesions of similar extent and intensity were observed in the skin of mice 15 min after the injection of 75 μg, 1.5 μg and 35 μg of the PI, PII and PIII SVMP, respectively, as expected when accounting for the highly different MHDs of these SVMPs. Homogenates of the sections of hemorrhagic skin were analyzed by immunoblot for the detection of several ECM components. With regard to the immunodetection of type IV collagen, samples from control skin injected with saline solution showed a predominant band of 107 kDa, with additional faint bands of 216 kDa, 176 kDa, 165 kDa, 117 kDa, and 97 kDa (Fig 3A). A conspicuous degradation band of 97 kDa was observed in samples from skins injected with the three SVMPs, together with a reduction in the intensity of the 107 kDa band (Fig 3A). The intensity of the 97 kDa band had the following order: PI > PII > PIII. Regarding type VI collagen, PI SVMP induced a reduction in the intensity of the predominant band of 216 kDa, whereas the PII and PIII SVMPs did not seem to hydrolyze this chain (Fig 3B). Degradation products of 160 kDa and 140 kDa were observed in samples from mice injected with PI SVMP, whereas the intensity of these bands in samples corresponding to the other two SVMPs was less pronounced (Fig 3B).

When laminin was immunodetected in skin homogenates, a predominant band of 167 kDa was observed, with additional bands of 270 kDa and 350 kDa (Fig 3C). The PI SVMP induced a greater hydrolysis of laminin, with the appearance of degradation products of 225 kDa and 50 kDa. The former band was not observed in samples from PII and PIII-treated mice (Fig 3C). The three SVMPs differ in their degradation of nidogen (Fig 3D). Samples from skin of mice injected with saline showed a predominant band of 135 kDa and few additional minor bands (Fig 3D). PI SVMP induced extensive degradation of nidogen, as evidenced by the disappearance of the 135 kDa band, and the appearance of a 47 kDa degradation band. A lower extent of hydrolysis was observed with the PII and PIII SVMPs. There was a reduction in the 135 kDa band, and the appearance of new bands of 100 kDa (in PIII SVMP) and of 47 kDa in samples injected with either enzyme (Fig 3D).

**Fig 4:**
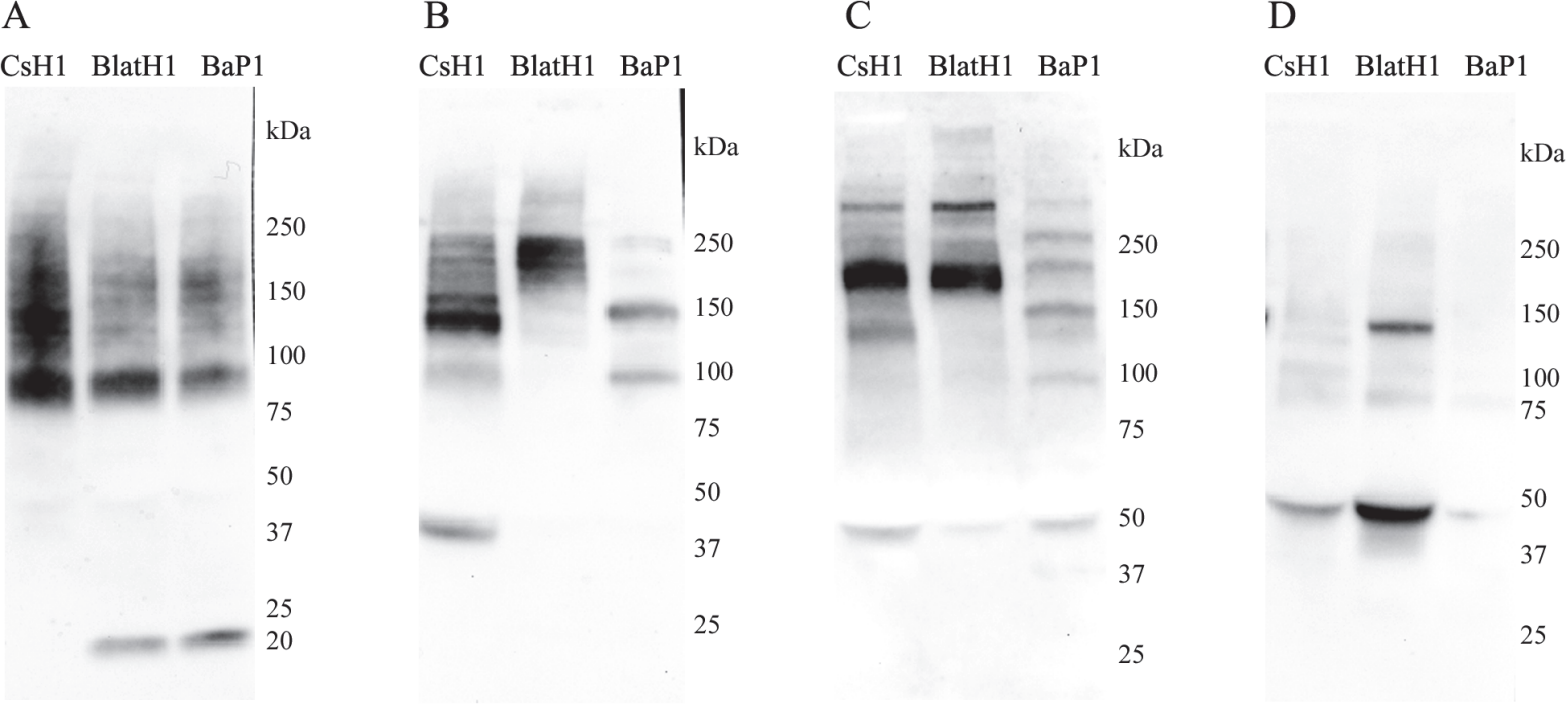
Western blot analysis of basement membrane components in exudates collected from the gastrocnemius. Groups of five mice were injected in the right gastrocnemius with either BaP1 (PI, 75 μg), BlatH1 (PII, 3 μg), or CsH1 (PIII, 50 μg) SVMPs. After 15 min, mice were sacrificed, a 5 mm incision was made in the skin overlying the injected muscle, and a heparinized capillary tube was introduced under the skin to collect the wound exudate fluid; exudate samples from a single treatment were then pooled. Afterwards, 100 μg of protein of each sample was separated under reducing conditions on 4–15% Tris–HCl SDS-PAGE, and transferred to nitrocellulose membranes. Immunodetection was performed with (A) anti-collagen type IV, (B) anti-collagen type VI, (C) anti-laminin, and (D) anti-nidogen 1. The reaction was detected using an anti-rabbit peroxidase antibody and a chemiluminescent substrate. Images were obtained with the ChemiDoc XRS+ System (BioRad).

Western blot analysis of exudates collected 15 min after injection of the enzymes revealed both similarities and differences between the three SVMPs. A relatively similar pattern was observed in the case of type IV collagen, with the presence of a predominant band of 90 kDa (Fig 4A). Samples from mice injected with PI and PII SVMPs showed a band of 20 kDa, which was not present in the case of PIII SVMP. In contrast, PIII SVMP generated a band of 140 kDa not observed in the case of the other two enzymes. A highly variable pattern of immunoreactivity was observed in exudates when tested for type VI collagen degradation products (Fig 4B). PI SVMP generated fragments of 225 kDa, 150 kDa, and 100 kDa. On the other hand, bands of 220 kDa and 200 kDa were observed in samples from PII SVMP-injected mice, and bands of 225 kDa, 200 kDa, 160 kDa, 140 kDa, and 40 kDa were present in exudates as a consequence of the action of PIII SVMP (Fig 4B). In the case of laminin, exudate from mice injected with PI SVMP showed bands of 275 kDa, 230 kDa, 200 kDa, 150 kDa, 105 kDa and 50 kDa (Fig 4C). On the other hand, PII and PIII SVMPs generated a similar pattern of immunoreactive bands of 275 kDa, 190 kDa, and 50 kDa, with an additional band of 140 kDa in the case of PIII SVMP (Fig 4C). Analysis of nidogen in exudates showed the presence of immunoreactive bands of 140 kDa and 50 kDa in the case of PII SVMP, and of 50 kDa in the case of PIII SVMP, whereas no immunoreactive bands were observed in the exudate of mice injected with PI SVMP (Fig 4D).

### Proteomic characterization of exudates

A similar protein pattern was observed when exudates were separated by 1D SDS-PAGE. From the mass spectral analysis of the gel bands, a total of 297, 354 and 322 proteins, with protein identification probability greater than 95% and minimum of two peptides, were identified in exudates collected from mice injected with PI, PII and PIII SVMPs, respectively (S1 Table includes the complete report of all the proteins identified in the exudates). The most abundant proteins identified based on their quantitative value (see http://www.proteomesoftware.com/ for full description of term) were analyzed. Proteins were classified within the following groups, and subgroups: (a) serum proteins; (b) proteins of the coagulation cascade; (c) proteinase inhibitors of plasma; (d) intracellular proteins; (e) keratins; (f) ECM proteins; and (g) membrane-associated proteins.

#### Serum proteins, including coagulation factors and proteinase inhibitors

S2–S4 Tables depict the quantitative values of serum proteins, proteins of the coagulation cascade and proteinase inhibitors, respectively. In general, similar values were observed for the vast majority of proteins in exudates collected from mice injected with the three SVMPs, especially in those with greatest quantitative values. Relatively minor differences were observed in the serum proteins (S2 Table). The highest variations occurred in fibrinogen, with higher amounts in the case of PI and PII SVMPs (S3 Table), and in some apolipoproteins, especially apolipoprotein B-100, of which the PI SVMP induced great amounts in the exudate (S2 Table). No relevant differences were noticed between SVMPs regarding the amounts of serum proteinase inhibitors (S4 Table).

#### Intracellular proteins

Similar amounts of hemoglobin chains occurred in exudates obtained from mice injected with the three SVMPs (S5 Table), as expected from the similar extent of hemorrhage induced. Likewise, similar amounts of many other proteins characterized the three types of exudates. However, there was a group of intracellular proteins in which higher values were detected in exudates from mice injected with PII and PIII SVMPs (S5 Table). Results on keratins are presented separately (S6 Table) owing to the relevance of skin damage induced by snake venoms. PII SVMP, and especially PI SVMP, induced a higher amount of keratins in exudates than PIII SVMP (S6 Table).

#### ECM proteins

As shown in Table 2, no differences were observed in detected BM proteins (heparin sulfate proteoglycan and nidogen). In contrast, there were differences in other ECM proteins. There was a tendency for higher values in several proteins in exudates collected from mice injected with the PI SVMP, such as tenascin, vitronectin, type VI collagen, type XIV collagen, type III collagen, and thrombospondin-4 (Table 2). Proteolysis of ECM proteins was analyzed on the basis of the range of molecular masses of the bands in SDS-PAGE gels. Identification of the various proteins in ranges of molecular mass lower than the known mass of the native proteins were considered degradation fragments, and the percentage of the total amount of each protein corresponding to hydrolyzed bands was estimated. As shown in Table 3, Results indicate that various types of collagens detected were degraded by the three SVMPs, with the exception of collagen type XIV, which was degraded 100% by the P-III, 33% by the PI and 0% by the PII. Regarding BM proteins, heparan sulfate proteoglycan and nidogen-1 were similarly degraded by the three SVMPs. Lumican was not degraded by any SVMP. Fibronectin was degraded to a higher extent by PI and PIII than by PII. Thrombospondin 4 was degraded only by the PI SVMP.

**Table 2:** Extracellular matrix proteins identified in wound exudate collected from mice injected with PI, PII or PIII SVMPs.

Protein	Accession Number	Mol. Mass	Quantitative value		
			P-I	P-II	P-III
Fibronectin	P11276	273 kDa	87	34	43
Tenascin X	E9Q2T3	340 kDa	**10**	0	0
Basement membrane-specific heparan sulfate proteoglycan core protein	B1B0C7 (+1)	469 kDa	4	3	2
Lumican	P51885	38 kDa	8	7	7
Vitronectin	P29788	55 kDa	**7**	5	2
Collagen alpha-1(I) chain	P11087	138 kDa	3	4	4
Protein Col6a3	E9PWQ3	354 kDa	**8**	1	2
Collagen alpha-1(XIV) chain	B7ZNH7 (+3)	193 kDa	**9**	3	2
Thrombospondin-4	Q9Z1T2	106 kDa	**4**	0	1
Nidogen-1	P10493	137 kDa	3	2	2
Collagen alpha-1(III) chain	P08121	139 kDa	**5**	1	3
Collagen alpha-1(XV) chain	A2AJY2 (+1)	138 kDa	0	0	**3**
Collagen alpha-2(I) chain	Q01149	130 kDa	**3**	0	**3**

Values in bold and underlined correspond to proteins for which at least one SVMP induced an increment of at least three times as compared to another SVMP.

**Table 3:** Degradation of extracellular matrix proteins identified in wound exudates (see Methods section for details).

Proteins	Accession number	Mol. mass	Percentage degradation		
			PI	PII	PIII
Fibronectin	P11276	273 kDa	45%	6%	76%
Tenascin X	E9Q2T3 (+1)	340 kDa	11%	^a^	^a^
BM-specific heparan sulfate proteoglycan core protein	B1B0C7 (+2)	469 kDa	50%	67%	50%
Vitronectin	P29788	55 kDa	100%	20%	50%
Lumican	P51885	38 kDa	0%	0%	0%
Thrombospondin-4	Q9Z1T2	106 kDa	75%	^a^	^a^
Nidogen-1	P10493	137 kDa	100%	100%	100%
Collagen alpha-1(XV) chain Col15a1	A2AJY2 (+3)	138 kDa	100%	^a^	100%
Collagen alpha-1(I) chain Col1a1	P11087	138 kDa	100%	100%	100%
Collagen alpha-1(III) chain Col3a1	P08121	139 kDa	100%	100%	100%
Collagen alpha-1(XIV) Col14a1	B7ZNH7 (+3)	193 kDa	33%	0%	100%
Collagen alpha-2(I) chain Col1a2	Q01149	130 kDa	100%	^a^	100%
Col6a3 (fragment)	D3YWD1 (+2)	186 kDa	100%	100%	^a^

^a^ Not detected.

#### Membrane-associated proteins

Seven membrane-associated proteins were detected in the exudates. With one exception, the amounts of these proteins did not differ more than 3-fold in exudates from mice injected with the three types of SVMPs (S7 Table).

## Discussion

This study analyzed, from a comparative perspective, the tissue localization and the degradation of ECM proteins and other plasma and cellular proteins in the tissues of mice injected with hemorrhagic PI, PII and PIII SVMPs. Since one of the main goals of this work was to gain further insights into the mechanisms of SVMP-induced microvessel damage leading to hemorrhage, the doses of SVMPs injected were standardized as to induce the same extent of hemorrhagic lesions. It was hypothesized that, in these experimental conditions, the ECM proteins whose hydrolysis is directly responsible for microvessel damage should be degraded to a similar extent by the three enzymes.

It has been proposed that one of the basis for the higher hemorrhagic activity of PIII SVMPs, as compared to PI enzymes, has to do with the ability of the former to locate in specific sites in microvasculature of tissues [9,13,18]. This has been demonstrated for the case of jararhagin, a PIII SVMP of the venom of *Bothrops jararaca*, where selective binding to microvessels and a pattern of co-localization of jararhagin and type IV collagen was described [8]. This selective binding is likely to depend on exosites located in the Dis-like and Cys-rich domains of PIII SVMPs. *In vitro* studies have demonstrated that PIII SVMPs selectively bind to proteins containing von Willebrand factor (vWF) A domains, such as vWF, fibrillar-associated collagens with interrupted triple helices (FACITs) and matrylins. Such interaction occurs between these proteins and sequences located in the Cys-rich domain of PIII SVMPs [8,11–14]. In addition, the Dis-like domain of jararhagin might contain sequences that mediate its binding with different types of collagen [9].

Our observations on the tissue localization of the three SVMPs in an *ex vivo* model conclusively demonstrate, using a quantitatively morphometric approach, the different pattern of distribution of PI and PIII SVMPs, since the former shows a more widespread pattern, whereas the latter preferentially bind to the microvessels and clearly co-localizes with type IV collagen. In addition, our study shows, for the first time, that a PII SVMP presents a pattern of distribution in the tissue highly similar to that of PIII enzymes, i.e. in the microvessels and co-localizing with type IV collagen. Hence, the dimeric PII SVMP, containing only metalloproteinase and disintegrin domains, is preferentially located in the microvasculature. This is reasonable given the high hemorrhagic activity of this PII SVMP [16]. The specific sequences in the PII and PIII enzymes mediating the interaction to microvessels, and the specific sites in the vasculature for interaction with SVMPs remain to be identified. The observed co-localization with type IV collagen does not exclude possible binding to other BM components; however, our findings support the concept that PII and PIII SVMPs preferentially co-localize with the BM. These results support the hypothesis that the high hemorrhagic activity of PII and PIII SVMPs is at least partially due to the selective localization of these enzymes in the BM of microvessels. The co-localization of PII and PIII SVMPs with type IV collagen was observed not only in capillary vessels, but also on arterioles and PCV, thus reflecting the localization of these SVMPs in the BM of the three types of microvessels. The possible pathological effects of SVMPs on these components of the microvasculature, in addition to capillaries, has not been studied and deserve consideration in order to fully understand the vascular pathology in snakebite envenoming.

One puzzling issue has to do with the large variation in hemorrhagic activity between the PII and PIII SVMPs, even though both present a similar pattern of distribution in the microvasculature. This finding agrees with previous observations with *Bothrops jararaca* hemorrhagic SVMPs in which there is a great difference in the hemorrhagic potential of PIII SVMPs [10]. These observations suggest that even though the high hemorrhagic activity of PII and PIII SVMPs largely depends on their ability to selectively bind to microvessels, other factors also determine the hemorrhagic potential of these enzymes. Differences in the turnover rate of hydrolysis of relevant substrates in the BM, especially of type IV collagen, may play a key role. Alternatively, PII and PIII SVMPs might present differences in the exosites in the Dis, Dis-like and Cys-rich domains, which determine subtle variations in the localization of these enzymes in the relevant substrates, with the consequent functional effects related to the proteolysis-induced mechanical destabilization of BM structure. Another possible explanation has to do with differences in the stability of these enzymes in the tissues, with more stable enzymes exerting a higher hemorrhagic effect. This subject deserves further investigation.

Since the hemorrhagic activity of SVMPs is likely to depend on the hydrolysis of BM components [8,18,22], particular attention was placed in this work to the analysis of degradation of BM proteins. Proteomics analysis of exudate collected in the vicinity of the hemorrhagic areas only detected perlecan and nidogen, and no differences were observed between the three SVMP classes regarding BM proteins. No protein fragments of laminin and type IV collagen were detected in this analysis. However, more sensitive immunochemical assessment of skin and exudates revealed subtle variations which might shed light on the mechanisms of hemorrhagic activity. Different patterns of hydrolysis were observed regarding nidogen, laminin and type VI collagen. In contrast, there were evident similarities between the three enzymes concerning hydrolysis of type IV collagen. This has interesting implications because a previous study identified type IV collagen as a likely candidate to play a key role in the onset of hemorrhagic activity, since hemorrhagic and non-hemorrhagic SVMPs differ in the extent of hydrolysis of this collagen [22]. Our present findings are therefore compatible with the hypothesis that degradation of type IV collagen is critical for microvessel damage and hemorrhage. This in turn agrees with the known role of this type of collagen in the mechanical stability of the BM [24–27,38], mostly owing to the presence of a covalently-linked network formed by this BM component [24]. Moreover, a genetic disease associated with mutations in the *COL4A1* gene and reduction in the expression of α1 subunit are associated with pathological alterations in microvessels and hemorrhage in mice, and have been linked to hemorrhagic stroke in humans [39]. In contrast to type IV collagen, hydrolysis of nidogen, laminin and type VI collagen by the three SVMPs showed differences both in the degradation patterns and in the intensity of the bands observed by immunoblotting of exudates, where the PI enzyme showed a more extensive degradation of these substrates. These observations, in the context of a similar extent of hemorrhage by the three SVMPs, suggest that the hydrolysis of nidogen, laminin and type VI collagen might not be directly associated with the onset of microvessel damage leading to hemorrhage but rather may be a general by-product of microvessel damage.

On the other hand, there were notorious differences in the amounts of other ECM proteins, which are not BM components, in the proteomic analysis of exudates collected from mice injected with the three toxins. In general, PI SVMP induced the appearance of higher amounts of various ECM proteins in exudates. This observation may be due to two factors: since a higher absolute amount of this enzyme was injected, owing to its lower hemorrhagic activity, there was a higher proteolytic activity in the tissue, thus resulting in higher hydrolysis. The higher extent of hydrolysis in the case of the PI SVMP observed by immunoblotting of exudates supports this hypothesis. On the other hand, the presence of exosites in PII and PIII SVMPs may contribute to their localization at specific targets in the ECM and on cell membranes, thus reducing the probability of these enzymes to act in a widespread fashion on ECM components. In contrast, the PI SVMP, being devoid of such exosites, would have less restriction to hydrolyze an ample spectrum of ECM substrates, as observed in our proteomics results.

As would be expected, the majority of ECM proteins detected in exudates corresponded to proteolytic fragments, on the basis of their molecular mass. The different pattern of hydrolysis by the SVMP of various ECM components, as detected by Western blot analysis, might be due to variations in the cleavage site preferences among these enzymes. Alternatively, this might depend on the presence of exosites in the non-metalloproteinase domains, which target these enzymes to different substrates in the ECM including BM or to different sequences in particular substrates, as observed by Serrano et al. [12,13].

In addition to ECM proteins, proteomic analysis of exudates allows the detection of serum, intracellular and membrane-associated proteins, and these findings may shed light on the pathological action of SVMPs from a broader perspective. Similar quantitative patterns of plasma-derived proteins were detected in exudates from mice injected with the three toxins. This seems logical, as the presence of these proteins is largely a consequence of overt microvessel damage by the action of these enzymes. Hence, extravasation of blood results in similar amounts of serum plasma proteins, and of hemoglobin as well. An exception to this general trend was observed with hydrolysis products of fibrinogen, which were in higher amounts in exudates from animals injected with the PI SVMP. Since this enzyme has fibrinolytic activity [30] and was injected in a higher dose than the other two SVMPs, this may have resulted in hydrolysis of the fibrin formed as a consequence of extravasation and clot formation.

When the amounts of intracellular proteins were compared in exudates, it was of interest that the PI SVMP, and also the PII SVMP, induced a higher amount of keratins than PIII SVMP. It is suggested that this reflects the ability of the former SVMPs to induce dermonecrosis and blistering which has been shown to be the case with this particular PI SVMP [40], but has not been previously explored for the PII SVMP. It is noteworthy that the dermotoxic action of the PII SVMP occurs when injected at a very low dose. On the other hand, there were several other intracellular proteins whose amounts were higher in exudates collected from mice injected with PII and PIII SVMPs. This suggests that these enzymes induce a higher cytotoxic activity in various cell types in the tissue. The three SVMPs induced a similar extent of skeletal muscle damage, as revealed by the similar amounts of the cytosolic muscle cell marker creatine kinase in exudates; it has been suggested that hemorrhagic SVMPs induce myotoxicity as a consequence of tissue ischemia [41]. The higher amounts of several intracellular markers in exudates collected from PII and PIII SVMPs-injected mice may be due to the targeting of these enzymes, through exosites present in the additional domains, to sites in the plasma membrane or in the vicinity of cells, a hypothesis that remains to be investigated.

In conclusion, our findings demonstrate that PII and PIII hemorrhagic SVMPs co-localize with type IV collagen in capillaries, PCVs and arterioles, whereas PI SVMP presents a more widespread localization in the tissue. This difference in tissue localization is likely to be one of the main reasons behind the higher hemorrhagic activity characteristic of PII and PIII SVMPs, as compared to enzymes of the PI class. Furthermore, immunochemical results support the hypothesis that hydrolysis of type IV collagen is likely to be a key event in SVMP-induced microvessel damage and destabilization leading to hemorrhage.

## Supporting Information

S1 Table List of all proteins identified in wound exudates collected from mice injected with PI (*B. asper*), PII (*B. lateralis*) and PIII (*C. simus*) SVMPs. (PDF)

S2 Table Serum proteins identified in wound exudates collected from mice injected with PI, PII or PIII SVMPs. (PDF)

S3 Table Coagulation factors identified in wound exudates collected from mice injected with PI, PII or PIII SVMPs. (PDF)

S4 Table Serum proteinase inhibitors identified in wound exudates collected from mice injected with PI, PII or PIII SVMPs. (PDF)

S5 Table Intracellular proteins identified in wound exudates collected from mice injected with PI, PII or PIII SVMPs. (PDF)

S6 Table Keratins identified in wound exudates collected from mice injected with PI, PII or PIII SVMPs. (PDF)

S7 Table Membrane proteins identified in wound exudates collected from mice injected with PI, PII or PIII SVMPs. (PDF)
