# Supplementary material for: Tissue Localization and Extracellular Matrix Degradation by PI, PII and PIII Snake Venom Metalloproteinases: Clues on the Mechanisms of Venom-Induced Hemorrhage
Source: PLoS Negl Trop Dis. 2015 Apr 24;9(4):e0003731. doi: 10.1371/journal.pntd.0003731 (PMC4409213; doi:10.1371/journal.pntd.0003731)
Supplement: S1 Table — (PDF) [file pntd.0003731.s001.pdf]

S1 Table. List of all proteins identified in wound exudates collected from mice injected with PI (*B. asper*), PII (*B. lateralis*) and PIII (*C. simus*) SVMPs.

Bio Sample	MS/MS Sample name	M.W.	Protein identification probability	Protein percentage of total spectra	Exclusive unique peptide count	Exclusive unique peptide count	Total spectrum count	Percentage sequence coverage
B_asper	Mudpit_v140219v02	28 kDa	100%	0.00%	2	2	3	10%
B_asper	Mudpit_v140219v02	29 kDa	100%	0.00%	1	1	1	5%
B_asper	Mudpit_v140219v02	28 kDa	100%	0.00%	2	2	2	10%
B_asper	Mudpit_v140219v02	32 kDa	11%	0.00%	0	0	0	0%
B_asper	Mudpit_v140219v02	53 kDa	44%	0.00%	1	1	1	2%
B_asper	Mudpit_v140219v02	21 kDa	99%	0.00%	1	1	1	6%
B_asper	Mudpit_v140219v02	42 kDa	98%	0.02%	0	0	17	21%
B_asper	Mudpit_v140219v02	42 kDa	100%	0.02%	4	4	20	28%
B_asper	Mudpit_v140219v02	42 kDa	80%	0.02%	0	0	17	21%
B_asper	Mudpit_v140219v02	48 kDa	95%	0.00%	1	1	1	3%
B_asper	Mudpit_v140219v02	22 kDa	99%	0.00%	1	1	1	5%
B_asper	Mudpit_v140219v02	27 kDa	100%	0.00%	1	1	3	10%
B_asper	Mudpit_v140219v02	24 kDa	99%	0.00%	0	0	1	9%
B_asper	Mudpit_v140219v02	24 kDa	100%	0.00%	0	0	1	9%
B_asper	Mudpit_v140219v02	46 kDa	100%	0.07%	6	10	70	36%
B_asper	Mudpit_v140219v02	46 kDa	100%	0.08%	6	13	83	39%
B_asper	Mudpit_v140219v02	46 kDa	100%	0.06%	2	3	55	29%
B_asper	Mudpit_v140219v02	57 kDa	100%	0.01%	5	7	10	10%
B_asper	Mudpit_v140219v02	37 kDa	100%	0.03%	8	16	30	33%
B_asper	Mudpit_v140219v02	55 kDa	100%	0.01%	3	4	6	5%
B_asper	Mudpit_v140219v02	166 kDa	100%	0.22%	0	0	220	38%
B_asper	Mudpit_v140219v02	167 kDa	100%	0.23%	1	2	224	40%
B_asper	Mudpit_v140219v02	47 kDa	100%	0.01%	5	5	10	23%
B_asper	Mudpit_v140219v02	12 kDa	100%	0.00%	1	1	1	12%
B_asper	Mudpit_v140219v02	53 kDa	100%	0.01%	4	5	5	13%
B_asper	Mudpit_v140219v02	52 kDa	100%	0.02%	9	10	18	25%
B_asper	Mudpit_v140219v02	31 kDa	100%	0.07%	17	24	71	56%
B_asper	Mudpit_v140219v02	11 kDa	100%	0.02%	2	2	21	19%

Bio Sample	MS/MS Sample name	M.W.	Protein identification probability	Protein percentage of total spectra	Exclusive unique peptide count	Exclusive unique peptide count	Total spectrum count	Percentage sequence coverage
B_asper	Mudpit_v140219v02	45 kDa	100%	0.02%	10	10	20	27%
B_asper	Mudpit_v140219v02	504 kDa	100%	0.02%	14	15	23	3%
B_asper	Mudpit_v140219v02	22 kDa	100%	0.00%	2	3	4	13%
B_asper	Mudpit_v140219v02	36 kDa	100%	0.04%	10	16	37	35%
B_asper	Mudpit_v140219v02	46 kDa	100%	0.00%	3	3	3	10%
B_asper	Mudpit_v140219v02	469 kDa	100%	0.00%	0	0	4	1%
B_asper	Mudpit_v140219v02	39 kDa	100%	0.01%	4	4	6	12%
B_asper	Mudpit_v140219v02	47 kDa	100%	0.02%	7	11	20	31%
B_asper	Mudpit_v140219v02	16 kDa	100%	0.15%	4	8	147	90%
B_asper	Mudpit_v140219v02	30 kDa	100%	0.01%	3	3	5	23%
B_asper	Mudpit_v140219v02	25 kDa	100%	0.00%	2	2	2	18%
B_asper	Mudpit_v140219v02	52 kDa	98%	0.00%	0	0	0	0%
B_asper	Mudpit_v140219v02	39 kDa	100%	0.00%	3	3	3	10%
B_asper	Mudpit_v140219v02	17 kDa	100%	0.00%	1	1	1	11%
B_asper	Mudpit_v140219v02	25 kDa	100%	0.00%	2	2	3	12%
B_asper	Mudpit_v140219v02	28 kDa	100%	0.01%	4	6	6	17%
B_asper	Mudpit_v140219v02	29 kDa	100%	0.01%	7	10	11	37%
B_asper	Mudpit_v140219v02	29 kDa	100%	0.01%	7	7	9	39%
B_asper	Mudpit_v140219v02	61 kDa	100%	0.07%	7	13	65	30%
B_asper	Mudpit_v140219v02	52 kDa	100%	0.01%	3	3	5	8%
B_asper	Mudpit_v140219v02	60 kDa	100%	0.01%	4	5	5	9%
B_asper	Mudpit_v140219v02	60 kDa	100%	0.00%	2	2	2	5%
B_asper	Mudpit_v140219v02	124 kDa	100%	0.12%	0	0	115	40%
B_asper	Mudpit_v140219v02	124 kDa	100%	0.12%	1	1	118	41%
B_asper	Mudpit_v140219v02	192 kDa	85%	0.00%	1	1	1	1%
B_asper	Mudpit_v140219v02	52 kDa	99%	0.00%	1	1	1	4%
B_asper	Mudpit_v140219v02	55 kDa	100%	0.00%	1	1	1	2%
B_asper	Mudpit_v140219v02	66 kDa	100%	0.01%	2	3	10	4%
B_asper	Mudpit_v140219v02	19 kDa	74%	0.00%	0	0	2	7%
B_asper	Mudpit_v140219v02	19 kDa	49%	0.00%	0	0	2	7%

Bio Sample	MS/MS Sample name	M.W.	Protein identification probability	Protein percentage of total spectra	Exclusive unique peptide count	Exclusive unique peptide count	Total spectrum count	Percentage sequence coverage
B_asper	Mudpit_v140219v02	138 kDa	100%	0.00%	3	3	3	3%
B_asper	Mudpit_v140219v02	139 kDa	100%	0.01%	3	5	5	3%
B_asper	Mudpit_v140219v02	193 kDa	100%	0.01%	7	8	9	5%
B_asper	Mudpit_v140219v02	138 kDa	100%	0.00%	0	0	0	0%
B_asper	Mudpit_v140219v02	130 kDa	100%	0.00%	3	3	3	3%
B_asper	Mudpit_v140219v02	27 kDa	100%	0.00%	2	2	4	10%
B_asper	Mudpit_v140219v02	26 kDa	100%	0.00%	1	1	1	5%
B_asper	Mudpit_v140219v02	186 kDa	100%	0.41%	65	101	407	46%
B_asper	Mudpit_v140219v02	193 kDa	100%	0.02%	11	13	22	7%
B_asper	Mudpit_v140219v02	61 kDa	100%	0.00%	1	1	2	2%
B_asper	Mudpit_v140219v02	23 kDa	100%	0.00%	3	3	4	19%
B_asper	Mudpit_v140219v02	80 kDa	97%	0.01%	0	0	6	8%
B_asper	Mudpit_v140219v02	141 kDa	100%	0.05%	18	30	47	23%
B_asper	Mudpit_v140219v02	67 kDa	100%	0.01%	7	7	11	14%
B_asper	Mudpit_v140219v02	45 kDa	100%	0.00%	2	2	2	6%
B_asper	Mudpit_v140219v02	43 kDa	100%	0.06%	15	25	60	49%
B_asper	Mudpit_v140219v02	36 kDa	100%	0.00%	2	2	2	10%
B_asper	Mudpit_v140219v02	50 kDa	100%	0.01%	3	5	11	11%
B_asper	Mudpit_v140219v02	50 kDa	100%	0.01%	2	2	7	8%
B_asper	Mudpit_v140219v02	50 kDa	100%	0.00%	2	2	3	5%
B_asper	Mudpit_v140219v02	95 kDa	100%	0.00%	0	0	0	0%
B_asper	Mudpit_v140219v02	135 kDa	100%	0.01%	8	8	9	9%
B_asper	Mudpit_v140219v02	46 kDa	99%	0.00%	0	0	1	2%
B_asper	Mudpit_v140219v02	272 kDa	100%	0.01%	5	5	6	2%
B_asper	Mudpit_v140219v02	15 kDa	100%	0.00%	3	3	3	27%
B_asper	Mudpit_v140219v02	21 kDa	19%	0.00%	0	0	0	0%
B_asper	Mudpit_v140219v02	43 kDa	100%	0.00%	3	3	4	12%
B_asper	Mudpit_v140219v02	55 kDa	100%	0.12%	26	48	116	56%
B_asper	Mudpit_v140219v02	49 kDa	100%	0.12%	23	45	116	57%
B_asper	Mudpit_v140219v02	273 kDa	100%	0.08%	40	47	83	24%

Bio Sample	MS/MS Sample name	M.W.	Protein identification probability	Protein percentage of total spectra	Exclusive unique peptide count	Exclusive unique peptide count	Total spectrum count	Percentage sequence coverage
B_asper	Mudpit_v140219v02	22 kDa	100%	0.00%	1	1	1	5%
B_asper	Mudpit_v140219v02	39 kDa	100%	0.03%	2	2	28	40%
B_asper	Mudpit_v140219v02	24 kDa	100%	0.00%	1	1	2	10%
B_asper	Mudpit_v140219v02	47 kDa	91%	0.00%	0	0	3	7%
B_asper	Mudpit_v140219v02	86 kDa	100%	0.03%	13	17	32	20%
B_asper	Mudpit_v140219v02	23 kDa	11%	0.00%	0	0	2	6%
B_asper	Mudpit_v140219v02	26 kDa	97%	0.00%	1	1	3	11%
B_asper	Mudpit_v140219v02	24 kDa	100%	0.00%	2	3	3	15%
B_asper	Mudpit_v140219v02	22 kDa	100%	0.00%	2	3	4	14%
B_asper	Mudpit_v140219v02	25 kDa	100%	0.01%	3	3	5	22%
B_asper	Mudpit_v140219v02	36 kDa	100%	0.01%	2	2	8	30%
B_asper	Mudpit_v140219v02	35 kDa	100%	0.00%	2	2	2	6%
B_asper	Mudpit_v140219v02	8 kDa	100%	0.00%	2	2	2	46%
B_asper	Mudpit_v140219v02	97 kDa	100%	0.00%	0	0	1	1%
B_asper	Mudpit_v140219v02	51 kDa	95%	0.00%	1	1	1	2%
B_asper	Mudpit_v140219v02	37 kDa	100%	0.01%	4	5	7	19%
B_asper	Mudpit_v140219v02	71 kDa	79%	0.00%	0	0	1	2%
B_asper	Mudpit_v140219v02	39 kDa	100%	0.05%	14	25	48	41%
B_asper	Mudpit_v140219v02	69 kDa	100%	0.02%	7	7	17	20%
B_asper	Mudpit_v140219v02	85 kDa	100%	0.00%	0	0	2	3%
B_asper	Mudpit_v140219v02	83 kDa	100%	0.01%	2	2	6	10%
B_asper	Mudpit_v140219v02	15 kDa	100%	0.07%	7	15	70	63%
B_asper	Mudpit_v140219v02	16 kDa	100%	0.19%	2	7	189	90%
B_asper	Mudpit_v140219v02	16 kDa	100%	0.14%	6	14	137	86%
B_asper	Mudpit_v140219v02	16 kDa	6%	0.02%	0	0	16	7%
B_asper	Mudpit_v140219v02	51 kDa	100%	0.10%	23	40	101	44%
B_asper	Mudpit_v140219v02	59 kDa	100%	0.01%	7	9	13	15%
B_asper	Mudpit_v140219v02	14 kDa	100%	0.00%	0	0	1	7%
B_asper	Mudpit_v140219v02	11 kDa	100%	0.00%	2	3	3	21%
B_asper	Mudpit_v140219v02	34 kDa	89%	0.00%	1	1	1	3%

Bio Sample	MS/MS Sample name	M.W.	Protein identification probability	Protein percentage of total spectra	Exclusive unique peptide count	Exclusive unique peptide count	Total spectrum count	Percentage sequence coverage
B_asper	Mudpit_v140219v02	43 kDa	100%	0.02%	5	7	17	17%
B_asper	Mudpit_v140219v02	44 kDa	100%	0.02%	5	8	17	18%
B_asper	Mudpit_v140219v02	44 kDa	100%	0.01%	3	3	6	10%
B_asper	Mudpit_v140219v02	44 kDa	100%	0.00%	2	2	2	8%
B_asper	Mudpit_v140219v02	13 kDa	100%	0.00%	2	2	2	28%
B_asper	Mudpit_v140219v02	15 kDa	99%	0.00%	1	1	4	16%
B_asper	Mudpit_v140219v02	12 kDa	100%	0.03%	5	9	33	49%
B_asper	Mudpit_v140219v02	12 kDa	100%	0.00%	2	2	2	36%
B_asper	Mudpit_v140219v02	14 kDa	99%	0.00%	1	1	1	12%
B_asper	Mudpit_v140219v02	50 kDa	100%	0.01%	5	6	14	16%
B_asper	Mudpit_v140219v02	18 kDa	99%	0.00%	1	1	2	9%
B_asper	Mudpit_v140219v02	77 kDa	100%	0.03%	15	16	31	24%
B_asper	Mudpit_v140219v02	105 kDa	100%	0.02%	16	19	23	20%
B_asper	Mudpit_v140219v02	106 kDa	100%	0.01%	7	7	11	7%
B_asper	Mudpit_v140219v02	102 kDa	100%	0.01%	10	10	14	15%
B_asper	Mudpit_v140219v02	99 kDa	100%	0.00%	3	3	3	4%
B_asper	Mudpit_v140219v02	74 kDa	87%	0.00%	0	0	0	0%
B_asper	Mudpit_v140219v02	28 kDa	100%	0.01%	3	3	6	13%
B_asper	Mudpit_v140219v02	81 kDa	100%	0.01%	7	7	11	10%
B_asper	Mudpit_v140219v02	70 kDa	100%	0.02%	9	11	17	18%
B_asper	Mudpit_v140219v02	50 kDa	100%	0.02%	3	3	23	11%
B_asper	Mudpit_v140219v02	48 kDa	100%	0.09%	1	1	88	36%
B_asper	Mudpit_v140219v02	58 kDa	100%	0.01%	6	6	8	16%
B_asper	Mudpit_v140219v02	47 kDa	97%	0.01%	0	0	11	19%
B_asper	Mudpit_v140219v02	46 kDa	100%	0.01%	0	0	7	12%
B_asper	Mudpit_v140219v02	46 kDa	98%	0.01%	0	0	11	20%
B_asper	Mudpit_v140219v02	45 kDa	91%	0.01%	0	0	8	16%
B_asper	Mudpit_v140219v02	51 kDa	100%	0.01%	3	3	8	14%
B_asper	Mudpit_v140219v02	48 kDa	53%	0.00%	0	0	4	3%
B_asper	Mudpit_v140219v02	52 kDa	100%	0.01%	0	0	8	3%

Bio Sample	MS/MS Sample name	M.W.	Protein identification probability	Protein percentage of total spectra	Exclusive unique peptide count	Exclusive unique peptide count	Total spectrum count	Percentage sequence coverage
B_asper	Mudpit_v140219v02	48 kDa	100%	0.01%	1	1	8	8%
B_asper	Mudpit_v140219v02	49 kDa	75%	0.00%	0	0	3	6%
B_asper	Mudpit_v140219v02	57 kDa	99%	0.01%	0	0	5	4%
B_asper	Mudpit_v140219v02	56 kDa	100%	0.03%	1	2	28	23%
B_asper	Mudpit_v140219v02	53 kDa	100%	0.03%	0	0	30	27%
B_asper	Mudpit_v140219v02	66 kDa	100%	0.02%	2	3	17	4%
B_asper	Mudpit_v140219v02	71 kDa	100%	0.01%	2	3	9	3%
B_asper	Mudpit_v140219v02	63 kDa	100%	0.01%	1	2	6	3%
B_asper	Mudpit_v140219v02	62 kDa	100%	0.01%	2	2	9	7%
B_asper	Mudpit_v140219v02	60 kDa	72%	0.01%	0	0	5	5%
B_asper	Mudpit_v140219v02	60 kDa	99%	0.01%	0	0	5	5%
B_asper	Mudpit_v140219v02	53 kDa	99%	0.08%	0	0	79	31%
B_asper	Mudpit_v140219v02	37 kDa	68%	0.01%	0	0	5	4%
B_asper	Mudpit_v140219v02	40 kDa	100%	0.02%	8	8	17	26%
B_asper	Mudpit_v140219v02	37 kDa	100%	0.01%	3	5	10	11%
B_asper	Mudpit_v140219v02	38 kDa	100%	0.01%	4	4	8	12%
B_asper	Mudpit_v140219v02	26 kDa	100%	0.00%	2	2	2	8%
B_asper	Mudpit_v140219v02	21 kDa	92%	0.00%	0	0	1	6%
B_asper	Mudpit_v140219v02	37 kDa	100%	0.00%	3	3	4	12%
B_asper	Mudpit_v140219v02	209 kDa	100%	0.00%	2	2	3	1%
B_asper	Mudpit_v140219v02	26 kDa	100%	0.01%	3	3	9	14%
B_asper	Mudpit_v140219v02	165 kDa	100%	0.11%	17	23	107	33%
B_asper	Mudpit_v140219v02	162 kDa	100%	0.06%	5	5	62	20%
B_asper	Mudpit_v140219v02	17 kDa	100%	0.00%	2	2	2	21%
B_asper	Mudpit_v140219v02	14 kDa	100%	0.00%	3	3	4	29%
B_asper	Mudpit_v140219v02	137 kDa	100%	0.00%	2	2	3	2%
B_asper	Mudpit_v140219v02	17 kDa	89%	0.00%	0	0	1	6%
B_asper	Mudpit_v140219v02	30 kDa	100%	0.00%	1	1	2	6%
B_asper	Mudpit_v140219v02	12 kDa	100%	0.01%	5	6	8	54%
B_asper	Mudpit_v140219v02	18 kDa	100%	0.01%	5	5	6	30%

Bio Sample	MS/MS Sample name	M.W.	Protein identification probability	Protein percentage of total spectra	Exclusive unique peptide count	Exclusive unique peptide count	Total spectrum count	Percentage sequence coverage
B_asper	Mudpit_v140219v02	25 kDa	100%	0.00%	3	3	3	19%
B_asper	Mudpit_v140219v02	22 kDa	100%	0.00%	0	0	3	10%
B_asper	Mudpit_v140219v02	22 kDa	100%	0.01%	5	6	6	27%
B_asper	Mudpit_v140219v02	50 kDa	81%	0.00%	0	0	0	0%
B_asper	Mudpit_v140219v02	21 kDa	100%	0.00%	3	3	4	28%
B_asper	Mudpit_v140219v02	93 kDa	100%	0.01%	8	9	13	11%
B_asper	Mudpit_v140219v02	61 kDa	100%	0.00%	2	2	2	4%
B_asper	Mudpit_v140219v02	45 kDa	100%	0.01%	4	4	7	16%
B_asper	Mudpit_v140219v02	29 kDa	100%	0.01%	1	1	7	9%
B_asper	Mudpit_v140219v02	29 kDa	100%	0.01%	3	3	10	17%
B_asper	Mudpit_v140219v02	46 kDa	100%	0.00%	1	1	2	3%
B_asper	Mudpit_v140219v02	71 kDa	100%	0.01%	7	7	11	13%
B_asper	Mudpit_v140219v02	56 kDa	100%	0.01%	5	5	11	12%
B_asper	Mudpit_v140219v02	91 kDa	100%	0.06%	25	35	60	38%
B_asper	Mudpit_v140219v02	37 kDa	8%	0.00%	0	0	0	0%
B_asper	Mudpit_v140219v02	83 kDa	92%	0.00%	0	0	3	4%
B_asper	Mudpit_v140219v02	50 kDa	100%	0.00%	2	3	4	7%
B_asper	Mudpit_v140219v02	29 kDa	40%	0.00%	1	1	1	5%
B_asper	Mudpit_v140219v02	39 kDa	100%	0.00%	4	4	4	10%
B_asper	Mudpit_v140219v02	62 kDa	100%	0.03%	2	2	33	13%
B_asper	Mudpit_v140219v02	354 kDa	100%	0.01%	6	6	8	2%
B_asper	Mudpit_v140219v02	20 kDa	50%	0.00%	0	0	0	0%
B_asper	Mudpit_v140219v02	87 kDa	100%	0.07%	16	26	72	20%
B_asper	Mudpit_v140219v02	108 kDa	100%	0.53%	0	0	520	46%
B_asper	Mudpit_v140219v02	142 kDa	100%	0.01%	1	1	9	6%
B_asper	Mudpit_v140219v02	40 kDa	95%	0.00%	0	0	2	5%
B_asper	Mudpit_v140219v02	44 kDa	100%	0.01%	1	1	7	15%
B_asper	Mudpit_v140219v02	112 kDa	100%	0.01%	1	1	6	2%
B_asper	Mudpit_v140219v02	340 kDa	100%	0.01%	8	8	10	4%
B_asper	Mudpit_v140219v02	70 kDa	100%	0.01%	1	1	6	11%

Bio Sample	MS/MS Sample name	M.W.	Protein identification probability	Protein percentage of total spectra	Exclusive unique peptide count	Exclusive unique peptide count	Total spectrum count	Percentage sequence coverage
B_asper	Mudpit_v140219v02	32 kDa	76%	0.00%	1	1	1	3%
B_asper	Mudpit_v140219v02	62 kDa	93%	0.00%	0	0	2	2%
B_asper	Mudpit_v140219v02	24 kDa	32%	0.00%	0	0	0	0%
B_asper	Mudpit_v140219v02	23 kDa	100%	0.00%	1	1	1	9%
B_asper	Mudpit_v140219v02	23 kDa	100%	0.00%	1	1	1	7%
B_asper	Mudpit_v140219v02	53 kDa	58%	0.00%	0	0	0	0%
B_asper	Mudpit_v140219v02	43 kDa	100%	0.00%	2	2	2	7%
B_asper	Mudpit_v140219v02	47 kDa	100%	0.15%	17	32	152	58%
B_asper	Mudpit_v140219v02	47 kDa	100%	0.05%	3	3	54	27%
B_asper	Mudpit_v140219v02	47 kDa	100%	0.04%	0	0	37	6%
B_asper	Mudpit_v140219v02	77 kDa	100%	0.55%	3	7	541	68%
B_asper	Mudpit_v140219v02	69 kDa	100%	1.09%	52	170	1077	78%
B_asper	Mudpit_v140219v02	15 kDa	100%	0.01%	3	3	7	22%
B_asper	Mudpit_v140219v02	26 kDa	100%	0.01%	6	8	11	26%
B_asper	Mudpit_v140219v02	40 kDa	100%	0.01%	4	4	6	14%
B_asper	Mudpit_v140219v02	83 kDa	100%	0.00%	0	0	0	0%
B_asper	Mudpit_v140219v02	16 kDa	100%	0.01%	3	3	5	23%
B_asper	Mudpit_v140219v02	12 kDa	100%	0.00%	2	2	2	21%
B_asper	Mudpit_v140219v02	106 kDa	100%	0.00%	2	3	4	3%
B_asper	Mudpit_v140219v02	48 kDa	100%	0.00%	1	1	1	3%
B_asper	Mudpit_v140219v02	86 kDa	100%	0.00%	2	2	2	3%
B_asper	Mudpit_v140219v02	89 kDa	100%	0.00%	1	1	2	2%
B_asper	Mudpit_v140219v02	68 kDa	100%	0.00%	1	1	1	4%
B_asper	Mudpit_v140219v02	16 kDa	100%	0.03%	5	6	34	47%
B_asper	Mudpit_v140219v02	32 kDa	100%	0.01%	1	1	12	17%
B_asper	Mudpit_v140219v02	50 kDa	57%	0.00%	0	0	2	4%
B_asper	Mudpit_v140219v02	50 kDa	41%	0.00%	0	0	2	4%
B_asper	Mudpit_v140219v02	18 kDa	90%	0.00%	0	0	3	19%
B_asper	Mudpit_v140219v02	17 kDa	95%	0.00%	1	1	1	7%
B_asper	Mudpit_v140219v02	19 kDa	86%	0.00%	0	0	0	0%

Bio Sample	MS/MS Sample name	M.W.	Protein identification probability	Protein percentage of total spectra	Exclusive unique peptide count	Exclusive unique peptide count	Total spectrum count	Percentage sequence coverage
B_asper	Mudpit_v140219v02	118 kDa	13%	0.00%	0	0	0	0%
B_asper	Mudpit_v140219v02	62 kDa	100%	0.00%	2	2	2	9%
B_asper	Mudpit_v140219v02	54 kDa	100%	0.07%	24	43	65	68%
B_asper	Mudpit_v140219v02	55 kDa	100%	0.01%	4	5	7	9%
B_asper	Mudpit_v140219v02	35 kDa	100%	0.00%	3	3	4	13%
B_lateralis	Mudpit_v140310v02	69 kDa	100%	1.07%	55	158	1095	80%
B_lateralis	Mudpit_v140310v02	77 kDa	100%	0.41%	2	4	420	68%
B_lateralis	Mudpit_v140310v02	108 kDa	100%	0.39%	0	0	403	46%
B_lateralis	Mudpit_v140310v02	186 kDa	100%	0.29%	59	83	299	46%
B_lateralis	Mudpit_v140310v02	16 kDa	100%	0.25%	7	18	254	86%
B_lateralis	Mudpit_v140310v02	16 kDa	100%	0.32%	4	7	329	90%
B_lateralis	Mudpit_v140310v02	16 kDa	100%	0.36%	3	7	365	90%
B_lateralis	Mudpit_v140310v02	16 kDa	98%	0.02%	1	1	22	12%
B_lateralis	Mudpit_v140310v02	167 kDa	100%	0.17%	1	2	176	38%
B_lateralis	Mudpit_v140310v02	166 kDa	100%	0.17%	0	0	172	37%
B_lateralis	Mudpit_v140310v02	31 kDa	100%	0.12%	18	34	120	55%
B_lateralis	Mudpit_v140310v02	46 kDa	100%	0.08%	6	13	85	37%
B_lateralis	Mudpit_v140310v02	46 kDa	100%	0.07%	5	7	70	31%
B_lateralis	Mudpit_v140310v02	46 kDa	100%	0.06%	2	2	62	27%
B_lateralis	Mudpit_v140310v02	47 kDa	100%	0.15%	15	27	152	46%
B_lateralis	Mudpit_v140310v02	47 kDa	100%	0.03%	0	0	35	16%
B_lateralis	Mudpit_v140310v02	47 kDa	100%	0.02%	2	2	25	11%
B_lateralis	Mudpit_v140310v02	15 kDa	100%	0.19%	8	20	190	69%
B_lateralis	Mudpit_v140310v02	165 kDa	100%	0.06%	13	17	60	24%
B_lateralis	Mudpit_v140310v02	162 kDa	100%	0.04%	8	8	39	19%
B_lateralis	Mudpit_v140310v02	51 kDa	100%	0.07%	22	34	73	46%
B_lateralis	Mudpit_v140310v02	43 kDa	100%	0.06%	14	22	64	48%
B_lateralis	Mudpit_v140310v02	48 kDa	100%	0.09%	1	1	87	45%
B_lateralis	Mudpit_v140310v02	53 kDa	100%	0.08%	1	1	83	41%
B_lateralis	Mudpit_v140310v02	124 kDa	100%	0.07%	1	1	73	38%

Bio Sample	MS/MS Sample name	M.W.	Protein identification probability	Protein percentage of total spectra	Exclusive unique peptide count	Exclusive unique peptide count	Total spectrum count	Percentage sequence coverage
B_lateralis	Mudpit_v140310v02	124 kDa	100%	0.07%	1	1	70	38%
B_lateralis	Mudpit_v140310v02	54 kDa	100%	0.07%	27	39	69	66%
B_lateralis	Mudpit_v140310v02	91 kDa	100%	0.04%	21	26	40	29%
B_lateralis	Mudpit_v140310v02	45 kDa	100%	0.07%	13	17	67	38%
B_lateralis	Mudpit_v140310v02	49 kDa	100%	0.05%	15	22	48	45%
B_lateralis	Mudpit_v140310v02	273 kDa	100%	0.03%	22	29	33	13%
B_lateralis	Mudpit_v140310v02	39 kDa	100%	0.03%	3	4	29	38%
B_lateralis	Mudpit_v140310v02	87 kDa	100%	0.03%	12	14	30	19%
B_lateralis	Mudpit_v140310v02	55 kDa	100%	0.03%	14	18	33	36%
B_lateralis	Mudpit_v140310v02	61 kDa	100%	0.06%	6	9	60	24%
B_lateralis	Mudpit_v140310v02	62 kDa	100%	0.03%	3	3	32	16%
B_lateralis	Mudpit_v140310v02	37 kDa	100%	0.05%	9	22	46	43%
B_lateralis	Mudpit_v140310v02	66 kDa	100%	0.03%	2	2	26	4%
B_lateralis	Mudpit_v140310v02	71 kDa	100%	0.01%	2	3	13	3%
B_lateralis	Mudpit_v140310v02	63 kDa	100%	0.01%	1	2	11	2%
B_lateralis	Mudpit_v140310v02	62 kDa	100%	0.01%	1	1	9	4%
B_lateralis	Mudpit_v140310v02	60 kDa	96%	0.00%	0	0	5	4%
B_lateralis	Mudpit_v140310v02	60 kDa	73%	0.00%	0	0	5	4%
B_lateralis	Mudpit_v140310v02	36 kDa	100%	0.07%	12	19	68	36%
B_lateralis	Mudpit_v140310v02	12 kDa	100%	0.03%	5	10	32	74%
B_lateralis	Mudpit_v140310v02	16 kDa	100%	0.03%	5	7	34	47%
B_lateralis	Mudpit_v140310v02	39 kDa	100%	0.02%	9	15	23	28%
B_lateralis	Mudpit_v140310v02	36 kDa	100%	0.03%	2	2	30	34%
B_lateralis	Mudpit_v140310v02	141 kDa	100%	0.02%	11	13	21	14%
B_lateralis	Mudpit_v140310v02	193 kDa	100%	0.03%	17	17	26	15%
B_lateralis	Mudpit_v140310v02	42 kDa	100%	0.05%	5	6	48	38%
B_lateralis	Mudpit_v140310v02	42 kDa	97%	0.04%	0	0	44	25%
B_lateralis	Mudpit_v140310v02	42 kDa	95%	0.04%	0	0	44	25%
B_lateralis	Mudpit_v140310v02	86 kDa	100%	0.03%	12	14	32	25%
B_lateralis	Mudpit_v140310v02	47 kDa	100%	0.02%	6	7	17	19%

Bio Sample	MS/MS Sample name	M.W.	Protein identification probability	Protein percentage of total spectra	Exclusive unique peptide count	Exclusive unique peptide count	Total spectrum count	Percentage sequence coverage
B_lateralis	Mudpit_v140310v02	47 kDa	8%	0.01%	0	0	6	7%
B_lateralis	Mudpit_v140310v02	29 kDa	100%	0.04%	10	17	43	59%
B_lateralis	Mudpit_v140310v02	57 kDa	100%	0.01%	8	8	14	21%
B_lateralis	Mudpit_v140310v02	340 kDa	100%	0.00%	0	0	0	0%
B_lateralis	Mudpit_v140310v02	142 kDa	100%	0.01%	0	0	14	6%
B_lateralis	Mudpit_v140310v02	80 kDa	100%	0.01%	0	0	10	9%
B_lateralis	Mudpit_v140310v02	40 kDa	100%	0.02%	7	7	16	26%
B_lateralis	Mudpit_v140310v02	50 kDa	100%	0.03%	3	3	30	13%
B_lateralis	Mudpit_v140310v02	48 kDa	100%	0.02%	2	2	16	11%
B_lateralis	Mudpit_v140310v02	52 kDa	100%	0.02%	1	1	19	8%
B_lateralis	Mudpit_v140310v02	48 kDa	96%	0.01%	0	0	10	8%
B_lateralis	Mudpit_v140310v02	52 kDa	100%	0.03%	9	10	27	23%
B_lateralis	Mudpit_v140310v02	106 kDa	100%	0.01%	7	7	15	8%
B_lateralis	Mudpit_v140310v02	22 kDa	100%	0.02%	7	7	19	36%
B_lateralis	Mudpit_v140310v02	11 kDa	100%	0.01%	1	1	6	10%
B_lateralis	Mudpit_v140310v02	70 kDa	100%	0.02%	10	12	21	20%
B_lateralis	Mudpit_v140310v02	105 kDa	100%	0.01%	11	11	15	13%
B_lateralis	Mudpit_v140310v02	43 kDa	100%	0.01%	5	6	15	17%
B_lateralis	Mudpit_v140310v02	77 kDa	100%	0.02%	8	10	19	14%
B_lateralis	Mudpit_v140310v02	71 kDa	100%	0.01%	6	6	11	12%
B_lateralis	Mudpit_v140310v02	97 kDa	100%	0.00%	0	0	1	1%
B_lateralis	Mudpit_v140310v02	29 kDa	100%	0.01%	5	6	9	26%
B_lateralis	Mudpit_v140310v02	67 kDa	100%	0.00%	4	4	4	8%
B_lateralis	Mudpit_v140310v02	44 kDa	100%	0.02%	5	9	23	20%
B_lateralis	Mudpit_v140310v02	12 kDa	100%	0.02%	6	6	18	65%
B_lateralis	Mudpit_v140310v02	93 kDa	100%	0.01%	5	5	9	6%
B_lateralis	Mudpit_v140310v02	70 kDa	100%	0.00%	1	2	5	9%
B_lateralis	Mudpit_v140310v02	32 kDa	100%	0.02%	2	2	21	31%
B_lateralis	Mudpit_v140310v02	37 kDa	100%	0.01%	5	7	11	26%
B_lateralis	Mudpit_v140310v02	40 kDa	71%	0.00%	0	0	4	13%

Bio Sample	MS/MS Sample name	M.W.	Protein identification probability	Protein percentage of total spectra	Exclusive unique peptide count	Exclusive unique peptide count	Total spectrum count	Percentage sequence coverage
B_lateralis	Mudpit_v140310v02	45 kDa	100%	0.01%	3	5	7	14%
B_lateralis	Mudpit_v140310v02	58 kDa	100%	0.01%	6	6	7	17%
B_lateralis	Mudpit_v140310v02	56 kDa	100%	0.00%	1	1	4	9%
B_lateralis	Mudpit_v140310v02	53 kDa	69%	0.00%	0	0	3	4%
B_lateralis	Mudpit_v140310v02	504 kDa	100%	0.00%	1	1	1	0%
B_lateralis	Mudpit_v140310v02	55 kDa	100%	0.01%	5	5	9	14%
B_lateralis	Mudpit_v140310v02	51 kDa	87%	0.00%	0	0	5	2%
B_lateralis	Mudpit_v140310v02	47 kDa	53%	0.00%	0	0	5	2%
B_lateralis	Mudpit_v140310v02	46 kDa	97%	0.00%	0	0	5	2%
B_lateralis	Mudpit_v140310v02	46 kDa	73%	0.00%	0	0	0	0%
B_lateralis	Mudpit_v140310v02	39 kDa	100%	0.01%	3	4	9	9%
B_lateralis	Mudpit_v140310v02	50 kDa	100%	0.01%	4	4	6	11%
B_lateralis	Mudpit_v140310v02	29 kDa	100%	0.01%	4	4	11	29%
B_lateralis	Mudpit_v140310v02	29 kDa	64%	0.00%	0	0	3	13%
B_lateralis	Mudpit_v140310v02	69 kDa	100%	0.01%	3	3	13	11%
B_lateralis	Mudpit_v140310v02	469 kDa	100%	0.00%	1	1	3	1%
B_lateralis	Mudpit_v140310v02	56 kDa	100%	0.01%	6	7	11	16%
B_lateralis	Mudpit_v140310v02	102 kDa	100%	0.01%	7	8	9	13%
B_lateralis	Mudpit_v140310v02	81 kDa	100%	0.01%	5	5	10	7%
B_lateralis	Mudpit_v140310v02	55 kDa	100%	0.00%	3	3	5	10%
B_lateralis	Mudpit_v140310v02	26 kDa	100%	0.01%	4	5	8	22%
B_lateralis	Mudpit_v140310v02	272 kDa	100%	0.00%	2	3	4	1%
B_lateralis	Mudpit_v140310v02	40 kDa	100%	0.00%	5	5	5	17%
B_lateralis	Mudpit_v140310v02	66 kDa	100%	0.01%	3	3	13	6%
B_lateralis	Mudpit_v140310v02	135 kDa	100%	0.00%	4	4	5	4%
B_lateralis	Mudpit_v140310v02	59 kDa	100%	0.01%	3	3	7	6%
B_lateralis	Mudpit_v140310v02	89 kDa	100%	0.01%	4	4	14	7%
B_lateralis	Mudpit_v140310v02	16 kDa	100%	0.01%	5	5	8	36%
B_lateralis	Mudpit_v140310v02	38 kDa	100%	0.01%	3	3	7	9%
B_lateralis	Mudpit_v140310v02	95 kDa	100%	0.01%	4	4	6	7%

Bio Sample	MS/MS Sample name	M.W.	Protein identification probability	Protein percentage of total spectra	Exclusive unique peptide count	Exclusive unique peptide count	Total spectrum count	Percentage sequence coverage
B_lateralis	Mudpit_v140310v02	44 kDa	100%	0.00%	0	0	0	0%
B_lateralis	Mudpit_v140310v02	52 kDa	100%	0.01%	7	8	9	20%
B_lateralis	Mudpit_v140310v02	15 kDa	100%	0.00%	1	1	4	14%
B_lateralis	Mudpit_v140310v02	30 kDa	100%	0.01%	3	4	10	25%
B_lateralis	Mudpit_v140310v02	17 kDa	95%	0.01%	0	0	6	18%
B_lateralis	Mudpit_v140310v02	43 kDa	100%	0.00%	4	4	4	20%
B_lateralis	Mudpit_v140310v02	50 kDa	100%	0.01%	2	3	13	12%
B_lateralis	Mudpit_v140310v02	47 kDa	100%	0.01%	5	5	14	20%
B_lateralis	Mudpit_v140310v02	28 kDa	100%	0.01%	5	6	7	31%
B_lateralis	Mudpit_v140310v02	83 kDa	95%	0.01%	0	0	6	5%
B_lateralis	Mudpit_v140310v02	18 kDa	97%	0.01%	0	0	6	24%
B_lateralis	Mudpit_v140310v02	50 kDa	100%	0.01%	1	2	7	16%
B_lateralis	Mudpit_v140310v02	50 kDa	74%	0.00%	0	0	4	10%
B_lateralis	Mudpit_v140310v02	22 kDa	100%	0.01%	7	8	8	49%
B_lateralis	Mudpit_v140310v02	60 kDa	100%	0.00%	2	2	3	3%
B_lateralis	Mudpit_v140310v02	50 kDa	100%	0.02%	2	2	16	36%
B_lateralis	Mudpit_v140310v02	50 kDa	100%	0.01%	1	1	14	29%
B_lateralis	Mudpit_v140310v02	138 kDa	100%	0.00%	3	3	4	4%
B_lateralis	Mudpit_v140310v02	83 kDa	100%	0.01%	6	6	10	11%
B_lateralis	Mudpit_v140310v02	52 kDa	100%	0.01%	2	3	6	6%
B_lateralis	Mudpit_v140310v02	99 kDa	100%	0.00%	3	3	3	4%
B_lateralis	Mudpit_v140310v02	18 kDa	100%	0.01%	5	5	6	21%
B_lateralis	Mudpit_v140310v02	354 kDa	100%	0.00%	1	1	1	0%
B_lateralis	Mudpit_v140310v02	28 kDa	100%	0.01%	3	5	7	19%
B_lateralis	Mudpit_v140310v02	60 kDa	100%	0.01%	3	3	6	7%
B_lateralis	Mudpit_v140310v02	28 kDa	100%	0.00%	1	1	3	8%
B_lateralis	Mudpit_v140310v02	28 kDa	100%	0.00%	2	2	4	13%
B_lateralis	Mudpit_v140310v02	32 kDa	99%	0.00%	1	1	3	8%
B_lateralis	Mudpit_v140310v02	24 kDa	100%	0.00%	0	0	0	0%
B_lateralis	Mudpit_v140310v02	24 kDa	6%	0.00%	0	0	0	0%

Bio Sample	MS/MS Sample name	M.W.	Protein identification probability	Protein percentage of total spectra	Exclusive unique peptide count	Exclusive unique peptide count	Total spectrum count	Percentage sequence coverage
B_lateralis	Mudpit_v140310v02	30 kDa	100%	0.00%	2	2	5	19%
B_lateralis	Mudpit_v140310v02	61 kDa	100%	0.00%	4	4	5	11%
B_lateralis	Mudpit_v140310v02	22 kDa	100%	0.01%	4	6	10	28%
B_lateralis	Mudpit_v140310v02	23 kDa	100%	0.01%	6	7	7	28%
B_lateralis	Mudpit_v140310v02	26 kDa	100%	0.00%	2	2	4	10%
B_lateralis	Mudpit_v140310v02	61 kDa	100%	0.00%	3	3	5	7%
B_lateralis	Mudpit_v140310v02	193 kDa	100%	0.00%	3	3	3	2%
B_lateralis	Mudpit_v140310v02	36 kDa	100%	0.00%	3	3	4	10%
B_lateralis	Mudpit_v140310v02	23 kDa	100%	0.00%	1	1	1	3%
B_lateralis	Mudpit_v140310v02	39 kDa	100%	0.00%	3	4	4	10%
B_lateralis	Mudpit_v140310v02	50 kDa	100%	0.01%	2	3	9	8%
B_lateralis	Mudpit_v140310v02	22 kDa	100%	0.00%	2	2	3	11%
B_lateralis	Mudpit_v140310v02	25 kDa	100%	0.00%	1	1	3	12%
B_lateralis	Mudpit_v140310v02	55 kDa	100%	0.00%	2	2	2	4%
B_lateralis	Mudpit_v140310v02	35 kDa	100%	0.00%	2	2	3	8%
B_lateralis	Mudpit_v140310v02	106 kDa	100%	0.00%	0	0	0	0%
B_lateralis	Mudpit_v140310v02	25 kDa	100%	0.00%	1	1	2	14%
B_lateralis	Mudpit_v140310v02	50 kDa	100%	0.00%	2	3	3	7%
B_lateralis	Mudpit_v140310v02	35 kDa	97%	0.00%	0	0	0	0%
B_lateralis	Mudpit_v140310v02	68 kDa	100%	0.00%	2	2	2	5%
B_lateralis	Mudpit_v140310v02	44 kDa	99%	0.00%	0	0	4	8%
B_lateralis	Mudpit_v140310v02	39 kDa	100%	0.00%	1	1	1	4%
B_lateralis	Mudpit_v140310v02	43 kDa	100%	0.00%	1	1	1	6%
B_lateralis	Mudpit_v140310v02	13 kDa	100%	0.00%	0	0	0	0%
B_lateralis	Mudpit_v140310v02	21 kDa	99%	0.00%	0	0	2	12%
B_lateralis	Mudpit_v140310v02	83 kDa	100%	0.01%	4	4	6	11%
B_lateralis	Mudpit_v140310v02	27 kDa	100%	0.00%	2	2	4	8%
B_lateralis	Mudpit_v140310v02	22 kDa	100%	0.01%	3	3	6	24%
B_lateralis	Mudpit_v140310v02	53 kDa	100%	0.00%	4	5	5	12%
B_lateralis	Mudpit_v140310v02	23 kDa	99%	0.00%	0	0	4	17%

Bio Sample	MS/MS Sample name	M.W.	Protein identification probability	Protein percentage of total spectra	Exclusive unique peptide count	Exclusive unique peptide count	Total spectrum count	Percentage sequence coverage
B_lateralis	Mudpit_v140310v02	26 kDa	90%	0.00%	0	0	4	15%
B_lateralis	Mudpit_v140310v02	15 kDa	100%	0.00%	1	1	1	9%
B_lateralis	Mudpit_v140310v02	137 kDa	100%	0.00%	2	2	2	2%
B_lateralis	Mudpit_v140310v02	21 kDa	100%	0.00%	3	3	3	19%
B_lateralis	Mudpit_v140310v02	21 kDa	100%	0.00%	1	1	1	8%
B_lateralis	Mudpit_v140310v02	24 kDa	100%	0.00%	3	3	4	20%
B_lateralis	Mudpit_v140310v02	21 kDa	100%	0.01%	3	3	6	25%
B_lateralis	Mudpit_v140310v02	50 kDa	100%	0.00%	3	3	3	8%
B_lateralis	Mudpit_v140310v02	46 kDa	100%	0.00%	0	0	2	2%
B_lateralis	Mudpit_v140310v02	139 kDa	100%	0.00%	1	1	1	1%
B_lateralis	Mudpit_v140310v02	29 kDa	100%	0.00%	2	2	2	9%
B_lateralis	Mudpit_v140310v02	37 kDa	100%	0.00%	2	2	3	8%
B_lateralis	Mudpit_v140310v02	138 kDa	100%	0.00%	0	0	0	0%
B_lateralis	Mudpit_v140310v02	26 kDa	100%	0.00%	3	3	3	18%
B_lateralis	Mudpit_v140310v02	112 kDa	99%	0.00%	1	1	4	1%
B_lateralis	Mudpit_v140310v02	24 kDa	100%	0.00%	3	3	4	20%
B_lateralis	Mudpit_v140310v02	37 kDa	100%	0.00%	4	4	4	14%
B_lateralis	Mudpit_v140310v02	45 kDa	100%	0.00%	0	0	0	0%
B_lateralis	Mudpit_v140310v02	23 kDa	100%	0.00%	2	2	3	15%
B_lateralis	Mudpit_v140310v02	48 kDa	97%	0.00%	1	1	1	3%
B_lateralis	Mudpit_v140310v02	51 kDa	100%	0.00%	3	3	4	7%
B_lateralis	Mudpit_v140310v02	32 kDa	100%	0.00%	2	2	2	11%
B_lateralis	Mudpit_v140310v02	52 kDa	100%	0.00%	2	2	2	4%
B_lateralis	Mudpit_v140310v02	53 kDa	100%	0.00%	1	1	4	6%
B_lateralis	Mudpit_v140310v02	17 kDa	100%	0.00%	1	1	4	21%
B_lateralis	Mudpit_v140310v02	19 kDa	98%	0.00%	0	0	2	11%
B_lateralis	Mudpit_v140310v02	20 kDa	100%	0.00%	1	1	3	30%
B_lateralis	Mudpit_v140310v02	46 kDa	100%	0.00%	3	3	3	12%
B_lateralis	Mudpit_v140310v02	101 kDa	100%	0.00%	2	2	4	3%
B_lateralis	Mudpit_v140310v02	22 kDa	98%	0.00%	1	1	2	6%

Bio Sample	MS/MS Sample name	M.W.	Protein identification probability	Protein percentage of total spectra	Exclusive unique peptide count	Exclusive unique peptide count	Total spectrum count	Percentage sequence coverage
B_lateralis	Mudpit_v140310v02	17 kDa	96%	0.00%	1	1	1	10%
B_lateralis	Mudpit_v140310v02	209 kDa	6%	0.00%	0	0	0	0%
B_lateralis	Mudpit_v140310v02	192 kDa	100%	0.00%	2	2	3	1%
B_lateralis	Mudpit_v140310v02	12 kDa	100%	0.00%	1	1	1	17%
B_lateralis	Mudpit_v140310v02	19 kDa	100%	0.00%	1	1	2	15%
B_lateralis	Mudpit_v140310v02	19 kDa	98%	0.00%	1	1	2	19%
B_lateralis	Mudpit_v140310v02	48 kDa	100%	0.00%	2	2	2	6%
B_lateralis	Mudpit_v140310v02	62 kDa	100%	0.00%	1	1	2	5%
B_lateralis	Mudpit_v140310v02	118 kDa	100%	0.00%	3	3	4	4%
B_lateralis	Mudpit_v140310v02	130 kDa	20%	0.00%	0	0	0	0%
B_lateralis	Mudpit_v140310v02	14 kDa	100%	0.00%	2	2	3	20%
B_lateralis	Mudpit_v140310v02	46 kDa	99%	0.00%	0	0	0	0%
B_lateralis	Mudpit_v140310v02	27 kDa	58%	0.00%	0	0	0	0%
B_lateralis	Mudpit_v140310v02	26 kDa	100%	0.00%	1	2	2	4%
B_lateralis	Mudpit_v140310v02	25 kDa	99%	0.00%	1	1	3	5%
B_lateralis	Mudpit_v140310v02	25 kDa	100%	0.00%	1	1	1	6%
B_lateralis	Mudpit_v140310v02	53 kDa	100%	0.00%	3	3	3	8%
B_lateralis	Mudpit_v140310v02	44 kDa	62%	0.00%	1	1	1	2%
B_lateralis	Mudpit_v140310v02	12 kDa	98%	0.00%	1	1	1	12%
B_lateralis	Mudpit_v140310v02	86 kDa	42%	0.00%	0	0	0	0%
B_lateralis	Mudpit_v140310v02	85 kDa	100%	0.00%	2	2	4	7%
B_lateralis	Mudpit_v140310v02	50 kDa	100%	0.00%	2	2	2	5%
B_lateralis	Mudpit_v140310v02	21 kDa	99%	0.00%	1	1	1	7%
B_lateralis	Mudpit_v140310v02	37 kDa	100%	0.00%	1	1	1	3%
B_lateralis	Mudpit_v140310v02	29 kDa	100%	0.00%	3	3	3	14%
B_lateralis	Mudpit_v140310v02	34 kDa	100%	0.00%	2	3	3	9%
B_lateralis	Mudpit_v140310v02	14 kDa	38%	0.00%	0	0	0	0%
B_lateralis	Mudpit_v140310v02	71 kDa	100%	0.00%	1	1	3	7%
B_lateralis	Mudpit_v140310v02	62 kDa	42%	0.00%	0	0	0	0%
B_lateralis	Mudpit_v140310v02	24 kDa	100%	0.00%	0	0	0	0%

Bio Sample	MS/MS Sample name	M.W.	Protein identification probability	Protein percentage of total spectra	Exclusive unique peptide count	Exclusive unique peptide count	Total spectrum count	Percentage sequence coverage
B_lateralis	Mudpit_v140310v02	37 kDa	98%	0.00%	0	0	4	8%
B_lateralis	Mudpit_v140310v02	18 kDa	100%	0.00%	3	3	3	18%
B_lateralis	Mudpit_v140310v02	14 kDa	100%	0.00%	2	2	2	26%
B_lateralis	Mudpit_v140310v02	8 kDa	94%	0.00%	0	0	0	0%
B_lateralis	Mudpit_v140310v02	23 kDa	100%	0.00%	2	2	2	10%
B_lateralis	Mudpit_v140310v02	17 kDa	100%	0.00%	2	2	2	14%
B_lateralis	Mudpit_v140310v02	23 kDa	100%	0.00%	1	1	2	10%
B_lateralis	Mudpit_v140310v02	22 kDa	100%	0.00%	2	2	2	12%
B_lateralis	Mudpit_v140310v02	22 kDa	100%	0.00%	3	3	3	18%
B_lateralis	Mudpit_v140310v02	23 kDa	100%	0.00%	2	2	2	12%
B_lateralis	Mudpit_v140310v02	21 kDa	100%	0.00%	1	1	2	11%
C_simus	Mudpit_v140312v05	69 kDa	100%	1.51%	55	178	1529	80%
C_simus	Mudpit_v140312v05	77 kDa	100%	0.52%	2	5	528	69%
C_simus	Mudpit_v140312v05	108 kDa	100%	0.50%	0	0	507	46%
C_simus	Mudpit_v140312v05	186 kDa	100%	0.33%	61	92	333	47%
C_simus	Mudpit_v140312v05	16 kDa	100%	0.16%	7	16	164	86%
C_simus	Mudpit_v140312v05	16 kDa	100%	0.23%	5	8	231	90%
C_simus	Mudpit_v140312v05	16 kDa	100%	0.27%	2	7	268	90%
C_simus	Mudpit_v140312v05	16 kDa	90%	0.01%	0	0	15	7%
C_simus	Mudpit_v140312v05	167 kDa	100%	0.22%	1	2	224	43%
C_simus	Mudpit_v140312v05	166 kDa	100%	0.22%	1	1	224	44%
C_simus	Mudpit_v140312v05	31 kDa	100%	0.13%	20	36	132	56%
C_simus	Mudpit_v140312v05	46 kDa	100%	0.11%	6	14	114	50%
C_simus	Mudpit_v140312v05	46 kDa	100%	0.08%	4	7	81	45%
C_simus	Mudpit_v140312v05	46 kDa	100%	0.08%	2	2	77	45%
C_simus	Mudpit_v140312v05	47 kDa	100%	0.18%	17	31	186	54%
C_simus	Mudpit_v140312v05	47 kDa	100%	0.04%	0	0	44	17%
C_simus	Mudpit_v140312v05	47 kDa	34%	0.03%	0	0	28	6%
C_simus	Mudpit_v140312v05	15 kDa	100%	0.19%	9	20	188	75%
C_simus	Mudpit_v140312v05	165 kDa	100%	0.06%	19	25	65	28%

Bio Sample	MS/MS Sample name	M.W.	Protein identification probability	Protein percentage of total spectra	Exclusive unique peptide count	Exclusive unique peptide count	Total spectrum count	Percentage sequence coverage
C_simus	Mudpit_v140312v05	162 kDa	100%	0.03%	2	2	28	10%
C_simus	Mudpit_v140312v05	51 kDa	100%	0.10%	22	40	96	48%
C_simus	Mudpit_v140312v05	43 kDa	100%	0.12%	17	28	118	53%
C_simus	Mudpit_v140312v05	48 kDa	100%	0.11%	2	2	116	46%
C_simus	Mudpit_v140312v05	53 kDa	100%	0.10%	0	0	97	38%
C_simus	Mudpit_v140312v05	124 kDa	100%	0.08%	1	1	77	40%
C_simus	Mudpit_v140312v05	124 kDa	100%	0.07%	1	1	72	41%
C_simus	Mudpit_v140312v05	54 kDa	100%	0.08%	23	38	80	68%
C_simus	Mudpit_v140312v05	91 kDa	100%	0.04%	23	24	42	33%
C_simus	Mudpit_v140312v05	45 kDa	100%	0.06%	13	15	56	43%
C_simus	Mudpit_v140312v05	49 kDa	100%	0.01%	3	3	6	7%
C_simus	Mudpit_v140312v05	273 kDa	100%	0.05%	25	30	46	15%
C_simus	Mudpit_v140312v05	39 kDa	100%	0.06%	3	7	59	64%
C_simus	Mudpit_v140312v05	87 kDa	100%	0.02%	7	7	23	12%
C_simus	Mudpit_v140312v05	55 kDa	100%	0.00%	3	3	3	8%
C_simus	Mudpit_v140312v05	61 kDa	100%	0.05%	7	12	52	27%
C_simus	Mudpit_v140312v05	62 kDa	100%	0.03%	2	2	27	13%
C_simus	Mudpit_v140312v05	37 kDa	100%	0.04%	8	21	45	41%
C_simus	Mudpit_v140312v05	66 kDa	100%	0.01%	1	1	9	4%
C_simus	Mudpit_v140312v05	71 kDa	96%	0.00%	0	0	0	0%
C_simus	Mudpit_v140312v05	63 kDa	95%	0.00%	1	1	1	2%
C_simus	Mudpit_v140312v05	62 kDa	64%	0.00%	0	0	1	2%
C_simus	Mudpit_v140312v05	60 kDa	83%	0.00%	0	0	1	2%
C_simus	Mudpit_v140312v05	60 kDa	66%	0.00%	0	0	1	2%
C_simus	Mudpit_v140312v05	36 kDa	100%	0.05%	12	17	55	37%
C_simus	Mudpit_v140312v05	12 kDa	100%	0.06%	5	12	61	48%
C_simus	Mudpit_v140312v05	16 kDa	100%	0.04%	7	10	44	63%
C_simus	Mudpit_v140312v05	39 kDa	100%	0.02%	9	13	23	28%
C_simus	Mudpit_v140312v05	36 kDa	100%	0.05%	3	5	47	48%
C_simus	Mudpit_v140312v05	141 kDa	100%	0.02%	9	10	16	11%

Bio Sample	MS/MS Sample name	M.W.	Protein identification probability	Protein percentage of total spectra	Exclusive unique peptide count	Exclusive unique peptide count	Total spectrum count	Percentage sequence coverage
C_simus	Mudpit_v140312v05	193 kDa	100%	0.02%	10	11	24	8%
C_simus	Mudpit_v140312v05	42 kDa	100%	0.02%	3	3	20	31%
C_simus	Mudpit_v140312v05	42 kDa	95%	0.02%	0	0	18	21%
C_simus	Mudpit_v140312v05	42 kDa	85%	0.02%	0	0	18	21%
C_simus	Mudpit_v140312v05	86 kDa	100%	0.02%	10	12	22	19%
C_simus	Mudpit_v140312v05	47 kDa	100%	0.03%	9	10	29	36%
C_simus	Mudpit_v140312v05	47 kDa	94%	0.01%	0	0	13	6%
C_simus	Mudpit_v140312v05	29 kDa	100%	0.02%	9	16	25	47%
C_simus	Mudpit_v140312v05	57 kDa	100%	0.04%	18	25	41	43%
C_simus	Mudpit_v140312v05	340 kDa	100%	0.00%	0	0	0	0%
C_simus	Mudpit_v140312v05	142 kDa	100%	0.01%	1	1	12	6%
C_simus	Mudpit_v140312v05	80 kDa	97%	0.01%	0	0	7	7%
C_simus	Mudpit_v140312v05	40 kDa	100%	0.03%	10	11	26	33%
C_simus	Mudpit_v140312v05	50 kDa	100%	0.01%	2	2	14	6%
C_simus	Mudpit_v140312v05	48 kDa	100%	0.00%	1	1	1	3%
C_simus	Mudpit_v140312v05	52 kDa	99%	0.00%	0	0	5	2%
C_simus	Mudpit_v140312v05	52 kDa	100%	0.02%	8	11	21	24%
C_simus	Mudpit_v140312v05	106 kDa	100%	0.02%	7	7	22	7%
C_simus	Mudpit_v140312v05	22 kDa	100%	0.02%	7	9	17	36%
C_simus	Mudpit_v140312v05	11 kDa	100%	0.04%	2	3	39	19%
C_simus	Mudpit_v140312v05	70 kDa	100%	0.02%	10	13	22	19%
C_simus	Mudpit_v140312v05	105 kDa	100%	0.02%	13	15	21	14%
C_simus	Mudpit_v140312v05	43 kDa	100%	0.01%	6	6	15	23%
C_simus	Mudpit_v140312v05	77 kDa	100%	0.03%	10	11	29	18%
C_simus	Mudpit_v140312v05	71 kDa	100%	0.01%	7	7	10	13%
C_simus	Mudpit_v140312v05	97 kDa	100%	0.01%	4	4	9	10%
C_simus	Mudpit_v140312v05	29 kDa	100%	0.02%	8	10	22	44%
C_simus	Mudpit_v140312v05	67 kDa	100%	0.00%	3	3	4	6%
C_simus	Mudpit_v140312v05	44 kDa	100%	0.03%	4	7	29	16%
C_simus	Mudpit_v140312v05	12 kDa	100%	0.03%	8	9	27	66%

Bio Sample	MS/MS Sample name	M.W.	Protein identification probability	Protein percentage of total spectra	Exclusive unique peptide count	Exclusive unique peptide count	Total spectrum count	Percentage sequence coverage
C_simus	Mudpit_v140312v05	93 kDa	100%	0.01%	6	7	10	8%
C_simus	Mudpit_v140312v05	70 kDa	100%	0.01%	2	2	6	11%
C_simus	Mudpit_v140312v05	32 kDa	100%	0.02%	1	1	24	36%
C_simus	Mudpit_v140312v05	37 kDa	100%	0.01%	5	6	9	18%
C_simus	Mudpit_v140312v05	40 kDa	97%	0.00%	0	0	3	8%
C_simus	Mudpit_v140312v05	45 kDa	100%	0.01%	4	5	12	15%
C_simus	Mudpit_v140312v05	58 kDa	100%	0.02%	10	10	18	25%
C_simus	Mudpit_v140312v05	56 kDa	100%	0.01%	1	1	9	14%
C_simus	Mudpit_v140312v05	53 kDa	100%	0.01%	0	0	9	15%
C_simus	Mudpit_v140312v05	504 kDa	100%	0.00%	1	1	1	0%
C_simus	Mudpit_v140312v05	55 kDa	100%	0.01%	6	6	8	20%
C_simus	Mudpit_v140312v05	51 kDa	100%	0.01%	3	3	6	9%
C_simus	Mudpit_v140312v05	49 kDa	97%	0.00%	0	0	5	5%
C_simus	Mudpit_v140312v05	47 kDa	100%	0.01%	0	0	6	9%
C_simus	Mudpit_v140312v05	46 kDa	46%	0.00%	0	0	5	5%
C_simus	Mudpit_v140312v05	46 kDa	66%	0.00%	0	0	3	7%
C_simus	Mudpit_v140312v05	45 kDa	8%	0.00%	0	0	5	5%
C_simus	Mudpit_v140312v05	39 kDa	100%	0.01%	5	6	11	14%
C_simus	Mudpit_v140312v05	50 kDa	100%	0.01%	4	4	11	11%
C_simus	Mudpit_v140312v05	29 kDa	100%	0.02%	6	8	16	32%
C_simus	Mudpit_v140312v05	29 kDa	34%	0.00%	0	0	2	9%
C_simus	Mudpit_v140312v05	69 kDa	100%	0.00%	2	2	5	6%
C_simus	Mudpit_v140312v05	469 kDa	100%	0.00%	0	0	2	1%
C_simus	Mudpit_v140312v05	56 kDa	100%	0.00%	3	3	4	10%
C_simus	Mudpit_v140312v05	102 kDa	100%	0.01%	4	4	6	8%
C_simus	Mudpit_v140312v05	81 kDa	100%	0.01%	4	5	7	7%
C_simus	Mudpit_v140312v05	55 kDa	100%	0.00%	1	1	2	3%
C_simus	Mudpit_v140312v05	26 kDa	100%	0.01%	7	10	12	27%
C_simus	Mudpit_v140312v05	272 kDa	100%	0.01%	5	5	9	2%
C_simus	Mudpit_v140312v05	40 kDa	100%	0.01%	4	5	6	17%

Bio Sample	MS/MS Sample name	M.W.	Protein identification probability	Protein percentage of total spectra	Exclusive unique peptide count	Exclusive unique peptide count	Total spectrum count	Percentage sequence coverage
C_simus	Mudpit_v140312v05	66 kDa	100%	0.00%	3	3	4	7%
C_simus	Mudpit_v140312v05	135 kDa	100%	0.00%	3	3	4	3%
C_simus	Mudpit_v140312v05	59 kDa	100%	0.01%	9	10	11	15%
C_simus	Mudpit_v140312v05	89 kDa	100%	0.01%	1	1	7	2%
C_simus	Mudpit_v140312v05	16 kDa	100%	0.00%	2	2	3	17%
C_simus	Mudpit_v140312v05	38 kDa	100%	0.01%	5	5	8	17%
C_simus	Mudpit_v140312v05	95 kDa	100%	0.00%	1	1	2	3%
C_simus	Mudpit_v140312v05	44 kDa	100%	0.01%	5	6	13	20%
C_simus	Mudpit_v140312v05	52 kDa	100%	0.01%	7	8	12	20%
C_simus	Mudpit_v140312v05	15 kDa	100%	0.01%	3	3	7	37%
C_simus	Mudpit_v140312v05	30 kDa	100%	0.00%	1	2	5	15%
C_simus	Mudpit_v140312v05	17 kDa	98%	0.00%	0	0	3	17%
C_simus	Mudpit_v140312v05	43 kDa	100%	0.01%	3	4	6	15%
C_simus	Mudpit_v140312v05	50 kDa	100%	0.01%	3	4	11	15%
C_simus	Mudpit_v140312v05	47 kDa	100%	0.01%	2	2	8	15%
C_simus	Mudpit_v140312v05	28 kDa	100%	0.01%	5	6	8	27%
C_simus	Mudpit_v140312v05	83 kDa	98%	0.00%	0	0	4	5%
C_simus	Mudpit_v140312v05	18 kDa	95%	0.00%	0	0	4	24%
C_simus	Mudpit_v140312v05	50 kDa	99%	0.00%	0	0	3	9%
C_simus	Mudpit_v140312v05	50 kDa	95%	0.00%	0	0	3	9%
C_simus	Mudpit_v140312v05	22 kDa	100%	0.01%	6	6	6	44%
C_simus	Mudpit_v140312v05	60 kDa	100%	0.01%	8	8	9	20%
C_simus	Mudpit_v140312v05	50 kDa	100%	0.01%	1	1	8	13%
C_simus	Mudpit_v140312v05	50 kDa	89%	0.01%	0	0	6	11%
C_simus	Mudpit_v140312v05	138 kDa	100%	0.00%	4	4	4	3%
C_simus	Mudpit_v140312v05	83 kDa	100%	0.00%	3	3	3	4%
C_simus	Mudpit_v140312v05	52 kDa	100%	0.00%	3	3	4	12%
C_simus	Mudpit_v140312v05	99 kDa	100%	0.00%	1	1	1	2%
C_simus	Mudpit_v140312v05	18 kDa	100%	0.00%	5	5	5	27%
C_simus	Mudpit_v140312v05	354 kDa	100%	0.00%	2	2	2	1%

Bio Sample	MS/MS Sample name	M.W.	Protein identification probability	Protein percentage of total spectra	Exclusive unique peptide count	Exclusive unique peptide count	Total spectrum count	Percentage sequence coverage
C_simus	Mudpit_v140312v05	28 kDa	100%	0.00%	3	4	4	19%
C_simus	Mudpit_v140312v05	60 kDa	100%	0.00%	2	2	4	5%
C_simus	Mudpit_v140312v05	28 kDa	100%	0.00%	1	1	3	8%
C_simus	Mudpit_v140312v05	28 kDa	100%	0.00%	1	1	3	7%
C_simus	Mudpit_v140312v05	24 kDa	100%	0.00%	1	1	5	20%
C_simus	Mudpit_v140312v05	24 kDa	100%	0.00%	1	1	5	17%
C_simus	Mudpit_v140312v05	30 kDa	100%	0.00%	1	1	3	11%
C_simus	Mudpit_v140312v05	61 kDa	100%	0.00%	2	2	2	4%
C_simus	Mudpit_v140312v05	22 kDa	100%	0.00%	4	5	5	37%
C_simus	Mudpit_v140312v05	23 kDa	100%	0.00%	4	5	5	36%
C_simus	Mudpit_v140312v05	26 kDa	100%	0.01%	2	2	8	11%
C_simus	Mudpit_v140312v05	57 kDa	100%	0.01%	4	4	13	10%
C_simus	Mudpit_v140312v05	61 kDa	100%	0.00%	3	3	5	7%
C_simus	Mudpit_v140312v05	193 kDa	100%	0.00%	1	2	2	1%
C_simus	Mudpit_v140312v05	36 kDa	100%	0.01%	2	2	9	10%
C_simus	Mudpit_v140312v05	23 kDa	100%	0.00%	4	4	5	30%
C_simus	Mudpit_v140312v05	39 kDa	100%	0.00%	1	1	1	3%
C_simus	Mudpit_v140312v05	50 kDa	100%	0.01%	2	2	9	12%
C_simus	Mudpit_v140312v05	22 kDa	100%	0.00%	1	1	1	7%
C_simus	Mudpit_v140312v05	25 kDa	100%	0.00%	2	2	3	18%
C_simus	Mudpit_v140312v05	55 kDa	100%	0.00%	4	4	4	9%
C_simus	Mudpit_v140312v05	35 kDa	100%	0.01%	6	7	7	22%
C_simus	Mudpit_v140312v05	106 kDa	100%	0.00%	1	1	1	2%
C_simus	Mudpit_v140312v05	25 kDa	100%	0.00%	3	3	5	30%
C_simus	Mudpit_v140312v05	50 kDa	100%	0.00%	2	3	4	7%
C_simus	Mudpit_v140312v05	35 kDa	100%	0.00%	2	2	2	6%
C_simus	Mudpit_v140312v05	68 kDa	94%	0.00%	0	0	0	0%
C_simus	Mudpit_v140312v05	44 kDa	100%	0.02%	2	2	16	24%
C_simus	Mudpit_v140312v05	39 kDa	100%	0.00%	3	4	4	10%
C_simus	Mudpit_v140312v05	43 kDa	100%	0.00%	1	1	1	6%

Bio Sample	MS/MS Sample name	M.W.	Protein identification probability	Protein percentage of total spectra	Exclusive unique peptide count	Exclusive unique peptide count	Total spectrum count	Percentage sequence coverage
C_simus	Mudpit_v140312v05	13 kDa	100%	0.00%	2	2	2	28%
C_simus	Mudpit_v140312v05	15 kDa	16%	0.00%	0	0	1	8%
C_simus	Mudpit_v140312v05	21 kDa	100%	0.00%	0	0	3	23%
C_simus	Mudpit_v140312v05	83 kDa	98%	0.00%	0	0	2	2%
C_simus	Mudpit_v140312v05	27 kDa	100%	0.01%	3	4	6	14%
C_simus	Mudpit_v140312v05	22 kDa	100%	0.00%	3	3	4	15%
C_simus	Mudpit_v140312v05	53 kDa	100%	0.00%	2	2	2	4%
C_simus	Mudpit_v140312v05	23 kDa	74%	0.00%	0	0	4	12%
C_simus	Mudpit_v140312v05	26 kDa	100%	0.00%	0	0	5	19%
C_simus	Mudpit_v140312v05	15 kDa	100%	0.00%	3	3	5	22%
C_simus	Mudpit_v140312v05	137 kDa	100%	0.00%	2	2	2	2%
C_simus	Mudpit_v140312v05	21 kDa	100%	0.00%	1	1	1	6%
C_simus	Mudpit_v140312v05	21 kDa	100%	0.00%	2	2	2	13%
C_simus	Mudpit_v140312v05	24 kDa	100%	0.00%	3	3	4	16%
C_simus	Mudpit_v140312v05	21 kDa	100%	0.00%	3	3	3	33%
C_simus	Mudpit_v140312v05	50 kDa	100%	0.00%	2	2	2	6%
C_simus	Mudpit_v140312v05	46 kDa	100%	0.00%	3	3	4	10%
C_simus	Mudpit_v140312v05	139 kDa	100%	0.00%	3	3	3	3%
C_simus	Mudpit_v140312v05	29 kDa	100%	0.00%	3	3	4	13%
C_simus	Mudpit_v140312v05	138 kDa	100%	0.00%	3	3	3	3%
C_simus	Mudpit_v140312v05	26 kDa	100%	0.00%	1	2	2	5%
C_simus	Mudpit_v140312v05	112 kDa	100%	0.00%	1	1	5	1%
C_simus	Mudpit_v140312v05	24 kDa	100%	0.00%	1	1	2	10%
C_simus	Mudpit_v140312v05	37 kDa	100%	0.00%	1	1	1	3%
C_simus	Mudpit_v140312v05	45 kDa	100%	0.00%	2	2	3	7%
C_simus	Mudpit_v140312v05	23 kDa	100%	0.00%	2	2	3	15%
C_simus	Mudpit_v140312v05	48 kDa	100%	0.00%	3	3	3	7%
C_simus	Mudpit_v140312v05	51 kDa	100%	0.00%	2	2	2	5%
C_simus	Mudpit_v140312v05	32 kDa	100%	0.00%	3	3	3	15%
C_simus	Mudpit_v140312v05	52 kDa	98%	0.00%	0	0	0	0%

Bio Sample	MS/MS Sample name	M.W.	Protein identification probability	Protein percentage of total spectra	Exclusive unique peptide count	Exclusive unique peptide count	Total spectrum count	Percentage sequence coverage
C_simus	Mudpit_v140312v05	53 kDa	98%	0.00%	0	0	1	2%
C_simus	Mudpit_v140312v05	17 kDa	98%	0.00%	1	1	1	11%
C_simus	Mudpit_v140312v05	19 kDa	76%	0.00%	0	0	0	0%
C_simus	Mudpit_v140312v05	20 kDa	100%	0.00%	1	1	3	31%
C_simus	Mudpit_v140312v05	46 kDa	100%	0.00%	2	2	2	10%
C_simus	Mudpit_v140312v05	101 kDa	12%	0.00%	0	0	0	0%
C_simus	Mudpit_v140312v05	22 kDa	100%	0.00%	2	3	3	13%
C_simus	Mudpit_v140312v05	17 kDa	100%	0.00%	1	2	2	10%
C_simus	Mudpit_v140312v05	209 kDa	7%	0.00%	0	0	0	0%
C_simus	Mudpit_v140312v05	12 kDa	100%	0.00%	2	3	5	36%
C_simus	Mudpit_v140312v05	19 kDa	99%	0.00%	0	0	0	0%
C_simus	Mudpit_v140312v05	19 kDa	99%	0.00%	1	1	1	8%
C_simus	Mudpit_v140312v05	14 kDa	100%	0.00%	1	1	2	20%
C_simus	Mudpit_v140312v05	48 kDa	100%	0.00%	3	3	3	8%
C_simus	Mudpit_v140312v05	62 kDa	37%	0.00%	0	0	1	2%
C_simus	Mudpit_v140312v05	118 kDa	22%	0.00%	0	0	0	0%
C_simus	Mudpit_v140312v05	130 kDa	100%	0.00%	3	3	3	3%
C_simus	Mudpit_v140312v05	14 kDa	97%	0.00%	1	1	1	10%
C_simus	Mudpit_v140312v05	46 kDa	100%	0.00%	1	1	1	3%
C_simus	Mudpit_v140312v05	13 kDa	100%	0.00%	2	2	4	13%
C_simus	Mudpit_v140312v05	27 kDa	100%	0.00%	1	1	2	9%
C_simus	Mudpit_v140312v05	74 kDa	99%	0.00%	0	0	2	3%
C_simus	Mudpit_v140312v05	26 kDa	96%	0.00%	1	1	1	8%
C_simus	Mudpit_v140312v05	25 kDa	100%	0.00%	2	2	4	12%
C_simus	Mudpit_v140312v05	25 kDa	100%	0.00%	3	3	3	22%
C_simus	Mudpit_v140312v05	44 kDa	100%	0.00%	2	2	2	7%
C_simus	Mudpit_v140312v05	11 kDa	100%	0.00%	1	1	1	10%
C_simus	Mudpit_v140312v05	12 kDa	100%	0.00%	2	2	3	24%
C_simus	Mudpit_v140312v05	86 kDa	11%	0.00%	0	0	0	0%
C_simus	Mudpit_v140312v05	85 kDa	20%	0.00%	0	0	2	2%

Bio Sample	MS/MS Sample name	M.W.	Protein identification probability	Protein percentage of total spectra	Exclusive unique peptide count	Exclusive unique peptide count	Total spectrum count	Percentage sequence coverage
C_simus	Mudpit_v140312v05	12 kDa	96%	0.00%	1	1	1	9%
C_simus	Mudpit_v140312v05	50 kDa	63%	0.00%	0	0	0	0%
C_simus	Mudpit_v140312v05	21 kDa	100%	0.00%	4	4	4	33%
C_simus	Mudpit_v140312v05	37 kDa	100%	0.00%	2	2	2	9%
C_simus	Mudpit_v140312v05	29 kDa	100%	0.00%	1	1	1	4%
C_simus	Mudpit_v140312v05	34 kDa	99%	0.00%	0	0	0	0%
C_simus	Mudpit_v140312v05	14 kDa	100%	0.00%	2	2	4	18%
C_simus	Mudpit_v140312v05	71 kDa	98%	0.00%	0	0	4	5%
C_simus	Mudpit_v140312v05	24 kDa	100%	0.00%	2	2	2	11%
C_simus	Mudpit_v140312v05	37 kDa	26%	0.00%	0	0	3	4%
C_simus	Mudpit_v140312v05	18 kDa	21%	0.00%	0	0	0	0%
C_simus	Mudpit_v140312v05	18 kDa	100%	0.00%	2	2	2	18%
C_simus	Mudpit_v140312v05	23 kDa	100%	0.00%	1	1	1	5%
C_simus	Mudpit_v140312v05	17 kDa	96%	0.00%	1	1	1	7%
C_simus	Mudpit_v140312v05	22 kDa	85%	0.00%	0	0	0	0%
C_simus	Mudpit_v140312v05	22 kDa	96%	0.00%	1	1	1	7%
C_simus	Mudpit_v140312v05	23 kDa	100%	0.00%	1	1	1	6%
C_simus	Mudpit_v140312v05	21 kDa	56%	0.00%	0	0	0	0%
C_simus	Mudpit_v140312v05	23 kDa	100%	0.00%	2	2	2	12%
