# Supplementary material for: Tissue Localization and Extracellular Matrix Degradation by PI, PII and PIII Snake Venom Metalloproteinases: Clues on the Mechanisms of Venom-Induced Hemorrhage
Source: PLoS Negl Trop Dis. 2015 Apr 24;9(4):e0003731. doi: 10.1371/journal.pntd.0003731 (PMC4409213; doi:10.1371/journal.pntd.0003731)
Supplement: S2 Table — (PDF) [file pntd.0003731.s002.pdf]

S2 Table. Serum proteins identified in wound exudates collected from mice injected with PI, PII or PIII SVMPs.

Proteins	Accession Number	Mol. Mass	Quantitative value		
			**P-I**	**P-II**	**P-III**
Serum albumin	P07724	69 kDa	1,126	1,132	1,421
Serotransferrin	Q921I1	77 kDa	565	434	491
Complement C3	P01027	186 kDa	425	309	309
Alpha-2-macroglobulin	D3YW52	167 kDa	234	182	208
Alpha-2-macroglobulin	Q61838	166 kDa	230	178	208
Apolipoprotein A-I	Q00623	31 kDa	74	124	123
Alpha-1-antitrypsin 1-3	Q00896	46 kDa	87	88	106
Alpha-1-antitrypsin 1-2	P22599	46 kDa	73	72	75
Alpha-1-antitrypsin 1-4	Q00897	46 kDa	57	64	71
Serine protease inhibitor A3K	P07759	47 kDa	159	157	173
Serine protease inhibitor A3M	Q03734	47 kDa	56	36	41
Serine protease inhibitor A3N	Q91WP6	47 kDa	39	26	26
Murinoglobulin-1	P28665	165 kDa	112	62	60
Murinoglobulin-2	P28666	162 kDa	65	40	26
Hemopexin	Q91X72	51 kDa	106	75	89
Isoform LMW of Kininogen-1	O08677-2	48 kDa	92	90	108
Kininogen-1	D3YTY9 (+2)	53 kDa	82	86	90
Ceruloplasmin	G3X8Q5	124 kDa	123	75	72
Ceruloplasmin	E9PZD8	124 kDa	120	72	67
Plasminogen	P20918	91 kDa	63	41	39
Apolipoprotein A-IV	P06728	45 kDa	21	**70**	52
Fibrinogen gamma chain	Q8VCM7	49 kDa	**121**	**50**	6
Fibronectin	P11276	273 kDa	87	34	43
Fibrinogen beta chain	Q8K0E8	55 kDa	**121**	**35**	3
Alpha-2-HS-glycoprotein	P29699	37 kDa	31	48	42
Ig kappa chain C region	P01837	12 kDa	34	33	57
Haptoglobin	Q61646	39 kDa	50	24	21
Complement factor H	E9Q8I0	141 kDa	**49**	22	14
Complement C4-B	P01029	193 kDa	23	27	22
Alpha-1B-glycoprotein	Q19LI2	57 kDa	10	14	**38**
Complement factor B	B8JJM6 (+1)	80 kDa	6	10	7
Antithrombin-III	P32261	52 kDa	19	28	19
Inter-alpha trypsin inhibitor, heavy chain 2	G3X977 (+1)	106 kDa	11	15	20
Apolipoprotein A-II	P09813	11 kDa	**22**	6	**36**
Inter alpha-trypsin inhibitor, heavy chain 4	A6X935 (+2)	105 kDa	24	15	19

Proteins	Accession Number	Mol. Mass	Quantitative value		
			P-I	P-II	P-III
Ig gamma-1 chain C region, membrane-bound form	P01869	43 kDa	18	15	14
Plasma kallikrein	P26262	71 kDa	11	11	9
Complement factor I	Q61129	67 kDa	11	4	4
Prothrombin	P19221	70 kDa	6	5	6
Apolipoprotein B-100 (Fragment)	E9Q1Y3	504 kDa	**24**	1	1
Alpha-2-antiplasmin	Q61247	55 kDa	6	9	7
Beta-2-glycoprotein 1	Q01339	39 kDa	6	9	10
Ig mu chain C region	P01872 (+1)	50 kDa	15	6	10
Plasma protease C1 inhibitor	P97290	56 kDa	11	11	4
Inter-alpha-trypsin inhibitor heavy chain H1	F8WJ05 (+1)	102 kDa	15	9	6
Vitronectin	P29788	55 kDa	**7**	**5**	2
Serum amyloid P-component	P12246	26 kDa	11	8	11
Serum paraoxonase/arylesterase 1	P52430	40 kDa	6	5	6
Coagulation factor XII	Q80YC5	66 kDa	10	13	4
Ig gamma-2B chain C region	P01867	44 kDa	**6**	0	**12**
Inter-alpha-trypsin inhibitor heavy chain H3	Q61704	99 kDa	3	3	1
Isoform 2 of Complement factor D	P03953-2	28 kDa	6	7	4
Alpha-1-acid glycoprotein 1	Q60590	24 kDa	1	0	**5**
Alpha-1-acid glycoprotein 2	P07361	24 kDa	1	0	**5**
Complement component C8 alpha chain	A2A998	61 kDa	2	5	2
Ig heavy chain V region 6.96	P18528	11 kDa	**3**	**3**	1
Ig heavy chain V region 914	P18527	11 kDa	**3**	**3**	0
Ig heavy chain V region MOPC 21 (Fragment)	P01783	15 kDa	**4**	0	1
Ig heavy chain V region 345	P18526	13 kDa	**3**	0	1
Complement C1q subcomponent subunit B	P14106	27 kDa	4	4	6
Complement C1q subcomponent subunit C	Q02105	26 kDa	1	**3**	2
Ig heavy chain V region PJ14	P01820	12 kDa	5	4	4
Apolipoprotein D	P51910	22 kDa	4	2	3
Ig kappa chain V-V region HP R16.7	P01644 (+1)	12 kDa	2	1	**5**

Values in bold and underlined correspond to proteins for which at least one SVMP induced an increment of at least three times as compared to another SVMP.
