# Supplementary material for: Tissue Localization and Extracellular Matrix Degradation by PI, PII and PIII Snake Venom Metalloproteinases: Clues on the Mechanisms of Venom-Induced Hemorrhage
Source: PLoS Negl Trop Dis. 2015 Apr 24;9(4):e0003731. doi: 10.1371/journal.pntd.0003731 (PMC4409213; doi:10.1371/journal.pntd.0003731)
Supplement: S3 Table — (PDF) [file pntd.0003731.s003.pdf]

S3 Table. Coagulation factors identified in wound exudates collected from mice injected with PI, PII or PIII SVMPs.

Proteins	Accession Number	Mol. Mass	Quantitative value		
			P-I	P-II	P-III
Fibrinogen gamma chain	Q8VCM7	49 kDa	**<u>121</u>**	**<u>50</u>**	6
Fibrinogen beta chain	Q8K0E8	55 kDa	**<u>121</u>**	**<u>35</u>**	3
Prothrombin	P19221	70 kDa	6	5	6
Coagulation factor XII	Q80YC5	66 kDa	10	**<u>13</u>**	4
Coagulation factor X	Q3U3V1	55 kDa	1	2	**<u>4</u>**

Values in bold and underlined correspond to proteins for which at least one SVMP induced an increment of at least three times as compared to another SVMP.
