# Supplementary material for: Tissue Localization and Extracellular Matrix Degradation by PI, PII and PIII Snake Venom Metalloproteinases: Clues on the Mechanisms of Venom-Induced Hemorrhage
Source: PLoS Negl Trop Dis. 2015 Apr 24;9(4):e0003731. doi: 10.1371/journal.pntd.0003731 (PMC4409213; doi:10.1371/journal.pntd.0003731)
Supplement: S4 Table — (PDF) [file pntd.0003731.s004.pdf]

S4 Table. Serum proteinase inhibitors identified in wound exudates collected from mice injected with PI, PII or PIII SVMPs.

Protein	Accession Number	Mol. Mass	Quantitative value		
			P-I	P-II	P-III
Alpha-2-macroglobulin	Q61838	166 kDa	230	178	208
Alpha-2-macroglobulin	D3YW52	167 kDa	234	182	208
Alpha-1-antitrypsin 1-3	Q00896	46 kDa	87	88	106
Alpha-1-antitrypsin 1-2	P22599	46 kDa	73	72	75
Alpha-1-antitrypsin 1-4	Q00897	46 kDa	57	64	71
Serine protease inhibitor A3K	P07759	47 kDa	159	157	173
Serine protease inhibitor A3M	Q03734	47 kDa	56	36	40
Serine protease inhibitor A3N	Q91WP6	47 kDa	39	26	26
Murinoglobulin-1	P28665	165 kDa	112	62	60
Murinoglobulin-2	P28666	162 kDa	65	40	26
Antithrombin-III	P32261	52 kDa	19	28	20
Inter-alpha trypsin inhibitor, heavy chain 2	G3X977 (+1)	106 kDa	11	15	20
Inter alpha-trypsin inhibitor, heavy chain 4	A6X935 (+2)	105 kDa	24	16	20
Alpha-2-antiplasmin	Q61247	55 kDa	6	9	7
Plasma protease C1 inhibitor	P97290	56 kDa	11	11	4
Inter-alpha-trypsin inhibitor heavy chain H1	F8WJ05 (+1)	102 kDa	15	9	6
Inter-alpha-trypsin inhibitor heavy chain H3	Q61704	99 kDa	<u>**3**</u>	<u>**3**</u>	1

Values in bold and underlined correspond to proteins for which at least one SVMP induced an increment of at least three times as compared to another SVMP.
