# Supplementary material for: Tissue Localization and Extracellular Matrix Degradation by PI, PII and PIII Snake Venom Metalloproteinases: Clues on the Mechanisms of Venom-Induced Hemorrhage
Source: PLoS Negl Trop Dis. 2015 Apr 24;9(4):e0003731. doi: 10.1371/journal.pntd.0003731 (PMC4409213; doi:10.1371/journal.pntd.0003731)
Supplement: S5 Table — (PDF) [file pntd.0003731.s005.pdf]

S5 Table. Intracellular proteins identified in wound exudates collected from mice injected with PI, PII or PIII SVMPs.

Protein	Accession Number	Mol. mass	Quantitative value		
			P-I	P-II	P-III
Hemoglobin subunit beta-2	P02089	16 kDa	143	263	152
Beta-globin	A8DUK4	16 kDa	154	340	215
Hemoglobin subunit beta-1	P02088	16 kDa	198	377	249
Hemoglobin subunit epsilon-Y2	P02104	16 kDa	17	23	14
Hemoglobin subunit alpha	P01942 (+1)	15 kDa	73	196	175
Creatine kinase M-type	P07310	43 kDa	63	66	110
Fructose-bisphosphate aldolase A	P05064	39 kDa	29	30	55
Carboxylesterase 1C	P23953	61 kDa	68	62	48
Glyceraldehyde-3-phosphate dehydrogenase	P16858	36 kDa	8	**31**	**44**
Actin, cytoplasmic 1	P60710	42 kDa	21	50	19
Actin, aortic smooth muscle	P62737	42 kDa	18	45	17
Actin, gamma-enteric smooth muscle	P63268	42 kDa	18	45	17
Gelsolin	P13020	86 kDa	33	33	20
Beta-enolase	P21550	47 kDa	21	18	27
Gamma-enolase	P17183	47 kDa	3	6	**12**
Carbonic anhydrase 2	P00920	29 kDa	11	**44**	23
L-lactate dehydrogenase	G5E8N5 (+1)	40 kDa	18	17	24
Glycogen phosphorylase, muscle form	Q9WUB3	97 kDa	1	1	**8**
Carbonic anhydrase 3	P16015	29 kDa	9	9	29
Parvalbumin alpha	P32848	12 kDa	8	19	25
Triosephosphate isomerase	P17751	32 kDa	12	22	22
Phosphoglycerate kinase 1	P09411	45 kDa	7	7	11
Phosphoglycerate kinase 2	P09041	45 kDa	3	2	4
Isoform M1 of Pyruvate kinase PKM	P52480-2	58 kDa	8	7	17
Phosphoglycerate mutase	O70250	29 kDa	10	11	15
Phosphoglycerate mutase 1	Q9DBJ1	29 kDa	**7**	3	2
Heat shock cognate 71 kDa protein	Q504P4	69 kDa	**18**	13	5
Satty acid synthase	P19096	272 kDa	6	4	8
DNA-dependent protein kinase catalytic subunit	P97313	471 kDa	2	1	2
E3 ubiquitin-protein ligase Zswim2	Q9D9X6	72 kDa	6	6	5
Transitional endoplasmic reticulum ATPase	Q01853	89 kDa	2	**14**	**7**
Superoxide dismutase [Cu-Zn]	P08228	16 kDa	5	8	3
Elongation factor 2	P58252	95 kDa	0	**6**	2
Clusterin	Q06890	52 kDa	1	**9**	**11**
Nucleoside diphosphate kinase	E9PZF0	30 kDa	2	**10**	5
Nucleoside diphosphate kinase A	P15532 (+1)	17 kDa	1	**6**	3

Protein	Accession Number	Mol. mass	Quantitative value		
			P-I	P-II	P-III
Elongation factor 1-alpha 1	P10126	50 kDa	11	13	10
Alpha-enolase	P17182	47 kDa	10	14	7
Carbonic anhydrase 1	P13634	28 kDa	6	7	7
Polyubiquitin-C	P0CG50	83 kDa	3	6	4
Ubiquitin-40S ribosomal protein S27a	P62983	18 kDa	3	6	4
Tubulin alpha-1C chain	P68373	50 kDa	2	**7**	3
Tubulin alpha-4A chain	P68368	50 kDa	2	4	3
Tubulin alpha-3 chain	P05214	50 kDa	2	5	3
Flavin reductase (NADPH)	Q923D2	22 kDa	1	**8**	**6**
Carboxypeptidase N subunit 2	Q9DBB9	60 kDa	5	3	8
Tubulin beta-4B chain	P68372	50 kDa	0	**16**	**7**
Tubulin beta-5 chain	P99024	50 kDa	0	**14**	**6**
Sulfhydryl oxidase 1	Q8BND5 (+1)	83 kDa	0	**10**	3
Carboxypeptidase N catalytic chain	Q9JJN5	52 kDa	5	6	4
Peptidyl-prolyl cis-trans isomerase A	P17742	18 kDa	6	6	5
Catalase	P24270	60 kDa	2	6	4
14-3-3 protein beta/alpha	Q9CQV8 (+1)	28 kDa	3	3	3
14-3-3 protein gamma	P61982	28 kDa	2	4	3
Bisphosphoglycerate mutase	P15327	30 kDa	5	5	3
Glutathione peroxidase 1	P11352	22 kDa	4	10	5
Phosphoglucomutase-1	Q9D0F9	61 kDa	2	5	5
Elongation factor 1-alpha 2	P62631	50 kDa	7	9	8
Adenylate kinase isoenzyme 1	Q9R0Y5 (+1)	22 kDa	1	**3**	1
Glutathione peroxidase 3	P46412	25 kDa	5	3	3
Peroxiredoxin 6	Q6GT24	25 kDa	3	2	5
Heat shock protein HSP 90-beta	P11499	83 kDa	**6**	**6**	2
Peroxiredoxin-1	P35700	22 kDa	3	6	4
Glutathione S-transferase Mu 1 (Fragment)	F6WHQ7	23 kDa	2	4	4
Glutathione S-transferase Mu 1	P10649	26 kDa	3	4	5
Glutathione S-transferase P 1	P19157	24 kDa	3	4	4
Phosphatidylethanolamine-binding protein 1	P70296	21 kDa	4	6	3
Elongation factor 1-gamma	Q9D8N0	50 kDa	3	3	2
Malate dehydrogenase, cytoplasmic	P14152	37 kDa	**4**	**4**	1
Rho GDP-dissociation inhibitor 1	Q99PT1	23 kDa	1	**3**	**3**
Guanine deaminase	Q9R111	51 kDa	1	**4**	2
Calmodulin	P62204 (+1)	17 kDa	1	**4**	1
C-1-tetrahydrofolate synthase, cytoplasmic	Q922D8	101 kDa	0	**4**	0
Alcohol dehydrogenase class-3	P28474	40 kDa	1	2	**5**
Maltase-glucoamylase	B5THE2	209 kDa	**3**	0	0

Protein	Accession Number	Mol. mass	Quantitative value		
			P-I	P-II	P-III
Clathrin heavy chain	Q5SXR6 (+1)	192 kDa	1	**<u>3</u>**	0
Ubiquitin-like modifier-activating enzyme 1	Q02053	118 kDa	0	**<u>4</u>**	0
Myosin light chain 1/3, skeletal muscle isoform (Fragment)	E0CZ30 (+3)	14 kDa	**<u>4</u>**	**<u>3</u>**	1
Aspartate aminotransferase, cytoplasmic	P05201	46 kDa	**<u>3</u>**	0	1
L-lactate dehydrogenase B chain	P16125	37 kDa	5	4	3

Values in bold and underlined correspond to proteins for which at least one SVMP induced an increment of at least three times as compared to another SVMP.
