# Supplementary material for: Tissue Localization and Extracellular Matrix Degradation by PI, PII and PIII Snake Venom Metalloproteinases: Clues on the Mechanisms of Venom-Induced Hemorrhage
Source: PLoS Negl Trop Dis. 2015 Apr 24;9(4):e0003731. doi: 10.1371/journal.pntd.0003731 (PMC4409213; doi:10.1371/journal.pntd.0003731)
Supplement: S6 Table — (PDF) [file pntd.0003731.s006.pdf]

S6 Table. Keratins identified in wound exudates collected from mice injected with PI, PII or PIII SVMPs.

Protein	Accession Number	Mol. Mass	Quantitative value		
			**P-I**	**P-II**	**P-III**
Keratin, type II cytoskeletal 1	P04104	66 kDa	18	**<u>27</u>**	8
Keratin, type II cytoskeletal 73	Q6NXH9	59 kDa	8	12	6
Keratin, type II cytoskeletal 2 epidermal	Q3TTY5	71 kDa	**<u>9</u>**	**<u>13</u>**	0
Keratin, type II cytoskeletal 2 oral	Q3UV17	63 kDa	**<u>6</u>**	**<u>11</u>**	1
Keratin, type II cytoskeletal 5	Q922U2	62 kDa	**<u>9</u>**	**<u>9</u>**	1
Keratin, type II cytoskeletal 6B	Q3UV11 (+1)	60 kDa	**<u>5</u>**	**<u>5</u>**	1
Keratin, type II cytoskeletal 75	Q8BGZ7	60 kDa	**<u>5</u>**	**<u>5</u>**	1
Isoform 3 of Keratin, type I cytoskeletal 10	P02535-3	50 kDa	24	31	13
Keratin, type I cytoskeletal 17	Q9QWL7	48 kDa	**<u>8</u>**	**<u>17</u>**	1
Keratin, type I cytoskeletal 16	Q9Z2K1	52 kDa	8	**<u>20</u>**	5
Keratin, type I cytoskeletal 13	P08730	48 kDa	**<u>4</u>**	**<u>10</u>**	0
Keratin, type II cuticular Hb5	Q9Z2T6	56 kDa	**<u>29</u>**	4	8
Keratin, type II cuticular Hb6	P97861	53 kDa	**<u>31</u>**	3	8
Keratin, type I cuticular Ha5	Q497I4	51 kDa	8	5	6
Keratin, type I cytoskeletal 40	Q6IFX3	49 kDa	**<u>3</u>**	0	**<u>4</u>**
Keratin, type I cuticular Ha1	Q61765	47 kDa	11	5	6
Keratin, type I cuticular Ha3-II	Q61897	46 kDa	11	5	5
Keratin, type I cuticular Ha3-I	Q8K0Y2	46 kDa	**<u>7</u>**	0	**<u>3</u>**
Keratin, type I cuticular Ha4	Q9D646	45 kDa	**<u>8</u>**	0	**<u>5</u>**
Keratin, type II cuticular Hb2	Q99M74	57 kDa	**<u>5</u>**	0	**<u>12</u>**

Values in bold and underlined correspond to proteins for which at least one SVMP induced an increment of at least three times as compared to another SVMP.
