# Supplementary material for: Tissue Localization and Extracellular Matrix Degradation by PI, PII and PIII Snake Venom Metalloproteinases: Clues on the Mechanisms of Venom-Induced Hemorrhage
Source: PLoS Negl Trop Dis. 2015 Apr 24;9(4):e0003731. doi: 10.1371/journal.pntd.0003731 (PMC4409213; doi:10.1371/journal.pntd.0003731)
Supplement: S7 Table — (PDF) [file pntd.0003731.s007.pdf]

S7 Table. Membrane proteins identified in wound exudates collected from mice injected with PI, PII or PIII SVMPs.

Proteins	Accession Number	Mol. Mass	Quantitative value		
			**P-I**	**P-II**	**P-III**
Ig gamma-1 chain C region, membrane-bound form	P01869	43 kDa	18	15	14
Ig gamma-2A chain C region, membrane-bound form	P01865	44 kDa	18	24	27
H-2 class I histocompatibility antigen, Q10 alpha chain	P01898	37 kDa	7	11	8
Isoform 2 of Leukemia inhibitory factor receptor	P42703-2	81 kDa	11	10	7
Epidermal growth factor receptor	Q01279	135 kDa	9	5	4
ATP-binding cassette sub-family A member 8-B	Q8K440 (+1)	183 kDa	3	5	2
Single-pass membrane and coiled-coil domain-containing protein 3	Q8BQM7	25 kDa	0	**<u>3</u>**	**<u>6</u>**

Values in bold and underlined correspond to proteins for which at least one SVMP induced an increment of at least three times as compared to another SVMP.
